# Association of preterm birth and birth size status with neurodevelopmental and psychiatric disorders in spontaneous births

**DOI:** 10.1007/s00787-024-02489-5

**Published:** 2024-06-12

**Authors:** Linghua Kong, Samson Nivins, Xinxia Chen, Yajun Liang, Mika Gissler, Catharina Lavebratt

**Affiliations:** 1 School of Nursing and Rehabilitation, Cheeloo College of Medicine, Shandong University, Shandong, China; 2 Department of Molecular Medicine and Surgery, Karolinska Institutet, Stockholm, Sweden; 3 Center for Molecular Medicine, Karolinska University Hospital, Stockholm, Sweden; 4 Department of Neuroscience, Karolinska Institutet, Stockholm, Sweden; 5 Department of Global Public Health, Karolinska Institutet, Stockholm, Sweden; 6 Department of Knowledge Brokers, Finnish Institute for Health and Welfare, Helsinki, Finland; 7 Translational Psychiatry Unit, Centre for Molecular Medicine, L8:00, Karolinska University Hospital, 171 76 Stockholm, Sweden

**Keywords:** Preterm birth, Birth size, Spontaneous birth, Psychiatric disorders, ADHD, Specific developmental disorders

## Abstract

Preterm birth (PTB) or small birth size are risk factors for certain neurodevelopmental disorders. The magnitude of these associations in spontaneous births, and of associations for combined PTB and birth size status on neurodevelopmental and psychiatric disorders is unexplored. We investigated whether PTB and small/large for gestational age (SGA/LGA), separately or combined, in spontaneous births, are associated with a wide spectrum of neurodevelopmental and psychiatric disorders. In this population-based registry cohort study, all singleton spontaneous births in Finland from 1996 to 2014 were followed until 2018 (n = 819 764). We show that PTB across gestational ages, and SGA, were associated with higher risks for anxiety disorders, intellectual disabilities, specific developmental disorders (SDD), autism spectrum disorders (ASD), attention-deficit/hyperactivity disorders (ADHD) and other emotional and behavioural disorders (F98). Most of these associations were not attributed to familial factors. Larger effect sizes were observed with lower gestational ages. Extremely PTB was associated at highest risks with intellectual disabilities (HR, 10.70 [95%CI, 8.69–13.17]) and SDD (HR, 8.91 [95%CI, 8.18–9.71]). Moreover, very preterm birth combined with SGA was associated with a higher risk for SDD (HR, 7.55 [95%CI, 6.61–8.62]) than that of very preterm or SGA birth alone. Conversely, LGA birth lowered the risk for SDD and other emotional and behavioural disorders among individuals born very preterm. In conclusion, PTB along with SGA is associated with higher risks for SDD than one exposure alone, whereas LGA lowers the risks for SDD and other emotional and behavioural disorders in individuals born spontaneously.

**Supplementary Information:**

The online version contains supplementary material available at 10.1007/s00787-024-02489-5.

## Introduction

Preterm birth (< 37 weeks; PTB) and/or small for gestational age (sex-specific birth weight and/or length < 2 SD for the gestational age; SGA) are the most common risk factors for neonatal morbidity and mortality [1, 2]. Both preterm and SGA births are at higher risk for neurodevelopmental disorders, such as attention-deficit/hyperactivity disorders (ADHD) during childhood and adolescence [3–10]. Moreover, these risks increase with a higher degree of severity of PTB or SGA [11, 12]. However, studies investigating the association between PTB or SGA and psychiatric disorders with onset commonly in late adolescence and early adulthood, such as depressive disorders, and anxieties, are limited [13, 14]. Furthermore, there is convincing evidence stating that PTB has a higher frequency of *in-utero* growth failures compared to term-born peers (> 37 weeks) [15], and PTB in combination with SGA, may pose more adverse sequelae. It is estimated that the global incidence of both preterm and SGA births is ~ 1.5 million [16], still, the magnitude of combined effects of PTB and SGA on neurodevelopmental and psychiatric disorders is under-explored.

Studies so far have shown a higher risk for a range of neurodevelopmental and psychiatric disorders in offspring born by caesarean delivery [17, 18]. However, there is limited information on the risk of psychiatric outcomes among those born preterm and SGA in spontaneous vaginal births.

Being born large for gestational age (LGA) has also, albeit less studied and less consistent than for SGA, been associated with a higher risk for the development of psychopathology [19, 20].

The risk of neurodevelopmental disorders, such as ADHD, is more prevalent in boys than girls [21, 22]. However, studies investigating the effect of sex on the relationship between PTB/SGA/LGA, and neurodevelopmental disorders are limited, especially in spontaneous births [23–25].

In addition, it remains unclear whether the observed association between the degree of prematurity or birth size status and the likelihood of developing neurodevelopmental or psychiatric disorders, as reported by earlier studies, might be confounded by unknown factors, such unmeasured shared familial factors, including genetics, household-level factors and lifestyle. Previous studies have utilized quasi-experimental designs, such as discordant sibling pairs, or maternal polygenic risk scores in attempts to control for these unmeasured familial factors [26–28].

We investigated the associations between birth outcomes, that is, PTB/SGA/LGA, separately and as combined risk, and a wide spectrum of neurodevelopmental and psychiatric disorders, and their psychotropic medication, in singleton individuals born of spontaneous births. We also conducted sibling pair analyses to investigate whether these associations could be explained by unmeasured familial confounding. Moreover, we investigated whether the association between birth outcomes and neurodevelopmental and psychiatric disorders differed by sex.

## Methods

### Study population and data sources

All live singleton spontaneous delivery births in Finland from 1996 to 2014 were included in this population-based registry cohort study (819 764 births including 299 331 sibling pairs), and follow-up until December 2018. All data were retrieved from nationwide registers: The Medical Birth Register (MBR), The Finnish Register on Reimbursement Drugs (RRD), and The Finnish Care Registers for Health Care (HILMO). Information from the different registers was linked using personal identification numbers (PIN) assigned to all Finnish citizens and permanent residents.

The complete study was approved by the relevant data protection authorities and ethical review committees in Finland and Sweden. According to Finnish regulations, informed consent by participants was not required, so the individuals included in this study were not contacted. This study followed the Strengthening the Reporting of Observational Studies in Epidemiology (STROBE) reporting guideline and data analysis was conducted from March 2021 to June 2023.

### Main exposures

Data on main exposures included gestational age in weeks and size for gestational age from MBR. Gestational age was assessed based on ultrasonography-based estimates at 11–13 weeks. The MBR collects information on the last menstrual period, which is cross-referenced in cases of suspected error in gestational age.

Gestational age was categorized as extremely preterm (< 28 weeks), very preterm (28–31 completed weeks), moderate-late preterm (32–36 completed weeks), term (37–41 completed weeks), and post-term (≥ 42 weeks). SGA/LGA was defined as birth weight and/or birth length, below/above two SDs from the gestation and sex-specific mean in the Finnish population [29], based on the International Societies of Pediatric Endocrinology and the Growth Hormone Research Society [30]. Children who were neither SGA nor LGA were considered appropriate for gestational age (AGA).

### Outcomes

We used *ICD-10* codes to ascertain neurodevelopmental and psychiatric disorders from HILMO in individuals between 1996 and 2018 (Table 1), and dispensation of psychotropic drugs (ATC codes N05 and N06) from RRD between 1996 and 2014.

**Table 1:** List of included neurodevelopmental and psychiatric disorders along with *ICD-10* codes, number of diagnosed children, and the corresponding proportions that were identified (until 2018) out of the estimated number of cases that would have received a diagnosis before 23 years of age (requiring the youngest cases to be followed up until 2036)

Neurodevelopmental and psychiatric disorders of interest	*ICD-10* codes	Number of diagnosed children	Estimated proportion identified (%)
Any neurodevelopmental or psychiatric disorder	F00-F99	137,163	
Psychotic disorders	F20-F29	2227	30.2
Mood disorders	F30-F39 and F92	32,361	46.4
Anxiety disorders	F40-F43 and F93	42,304	55.5
Eating disorders	F50	4939	43.0
Sleeping disorders	F51	5064	61.8
Personality disorders	F60-F69	2600	35.2
Intellectual disabilities	F70-F79	6379	86.5
SDD	F80-F83	44,720	92.5
ASD	F84	8719	88.6
ADHD	F90	21,879	99.2
Conduct disorders	F91	3715	55.2
Other behavioural and emotional disorders	F98	22,111	99.9

Estimated proportion identified was calculated as number of diagnosed cases divided by the denominator being: (proportion of diagnosed cases among those born in 1996 (this birth cohort was followed up for 22 years)*total cohort size 819,764)

The Finnish Care Registers for Health Care (HILMO) contains data on all hospital in-patient treatments (since 1969) as well as out-patient treatments by physicians in specialized care (since 1998) and includes all psychiatric diagnoses. The validity of paediatric ASD diagnoses in HILMO is good [31]

*SDD* specific developmental disorders; *ASD* autism spectrum disorders; *ADHD* attention-deficit/hyperactivity disorders

### Covariates

Based on a directed acyclic graph (Figure S1) the covariates were: birth year of the child, sex of the child (boy/girl), maternal age at child birth, parity (0 or ≥ 1), maternal cohabitation status at child birth (yes/no), maternal country of origin (Finland or other), maternal occupation (upper white-collar worker, lower white-collar worker, blue-collar worker, other status), smoking during pregnancy (yes/no) from MBR, and maternal obesity (*ICD-10* codes; E65-66, yes/no), maternal in-patient (from 1987) and out-patient (from 1998) psychiatric history (yes/no), maternal systemic inflammatory disease (*ICD-10* codes; M30-M36; yes/no) from MBR and HILMO, and maternal use of psychotropic medication during pregnancy (ATC codes; N05 or N06, yes/no) from RRD.

### Statistical analysis

Child and maternal characteristics were summarised according to birth outcomes (PTB/SGA/LGA). Baseline characteristics were compared between gestational groups and between birth size status groups using the χ^2^ test for categorical variables.

**Fig. 1:**
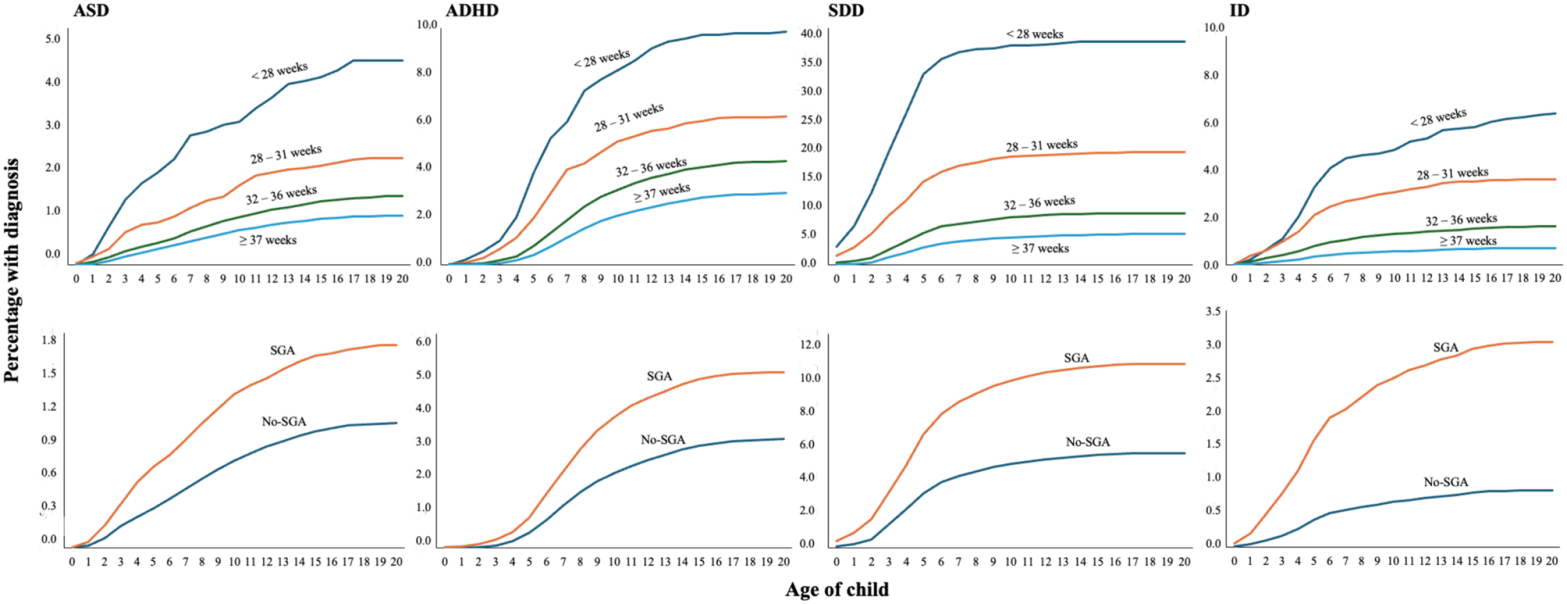
Cumulative incidence of autism spectrum disorder (ASD), ADHD, specific developmental disorder (SDD), intellectual disability (ID) across age of the born children stratified by exposure to preterm birth categories, or born small for gestational age (SGA)

We used Cox proportional hazards models to investigate the relationship between birth outcomes and neurodevelopmental and psychiatric disorders in individuals, unadjusted (crude) and after adjusting for confounders, listed above. The proportional hazards assumptions were tested mainly by evaluating the cumulative incidence of outcome in the different exposure groups across age of the child (Fig. 1). The single exposure analyses (PTB or SGA/LGA) disregarded information about a possible second exposure (PTB and SGA/LGA). Potential interactions between PTB and SGA/LGA were examined on multiplicative scales in a full-factor model with the aforementioned covariates. Due to the number of analyses conducted, and to control for Type 1 errors, a statistical significance threshold was set at p = 0.001 in the main covariate-adjusted analysis (= 0.05/50; Bonferroni-correction for 50 tests, based on 11 outcomes and 6 exposures that in-part are dependent on each other).

Sensitivity analyses were conducted to investigate (i) whether birth outcomes are associated with the individuals’ use of prescribed psychotropic drugs, (ii) sex-specific effect sizes, (iii) association with mood and anxiety diagnoses set from 10 years of age studying those followed 10 years or longer, and (iv) effect sizes of F98 subgroups. Here, two-sided *P* < 0.05 was considered statistically significant.

To account for possible confounding by shared familial factors, we conducted a sensitivity analysis comprising sibling pair analyses. The exposures were, due to sample sizes and effect sizes in the full cohort, limited to PTB and SGA. We estimated the risk of specific F-diagnosis in the second sibling, given the exposure or not exposure for the first and second siblings to birth outcomes. Unexposed first and second sibling pairs were used as references. We used two models of adjustments. In Model 1, we adjusted for all covariates as discussed above plus intra-pregnancy interval. In Model 2, we additionally adjusted for the presence of the corresponding F-diagnosis in the first sibling (yes/no) along with those covariates adjusted in Model 1.

Hazard risk ratios (HRs) with 95% confidence intervals (CIs) were reported as measures of effect size.

All statistical analyses were performed using SAS version 9.4 (SAS Institute Cary, USA).

## Results

Of the 819 764 singleton births (48.9% girls and 51.1% boys), 35 259 (4.3%) were born preterm, 21 977 (2.7%) were born post-term, 22 969 (2.8%) were born SGA, and 18 258 (2.2%) were born LGA. In our included cohort, 137 163 (16%) were diagnosed with a neurodevelopmental or psychiatric disorder between 1996 and 2018 (Table S1–S4).

In comparison to those born at term, individuals born preterm were more likely to have mothers who conceived at a younger age, were more likely to be obese before pregnancy, continued smoking during the whole pregnancy, had a history of psychiatric illness, and had lower socioeconomic status (occupation level) (Table S1). In addition, those born preterm were born smaller for gestational age compared to their term-born peers.

### Gestational age

**Fig. 2:**
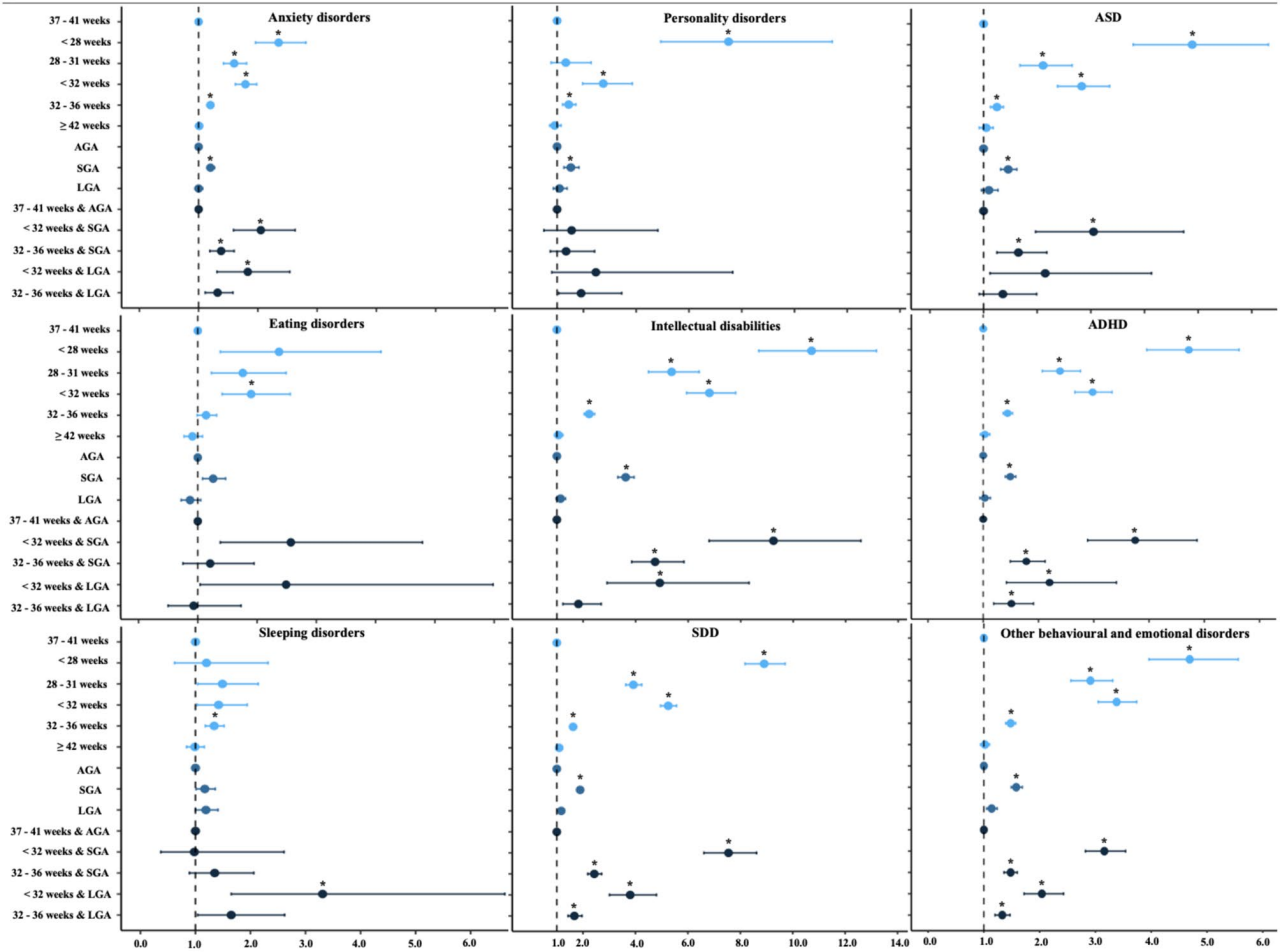
Adjusted hazard ratios (HRs) for neurodevelopmental and psychiatric disorders in relation to gestational age and size for gestational age (All live spontaneous singleton pregnancies born between 1996 and 2014 in Finland followed until 2018). Hazard ratios are represented on the x-axis, while gestational age and/or size for gestational age are represented on the y-axis. To achieve a more appropriate alignment of hazard ratios concerning intellectual disabilities, SDD, and personality disorders, we have plotted them on a separate x-axis range. *AGA* appropriate for gestational age; *SGA* small for gestational age; *LGA* large for gestational age; *SDD* specific developmental disorders; *ASD* autism spectrum disorders; *ADHD* attention-deficit/hyperactivity disorders. Extremely preterm is defined as < 28 weeks; very preterm is defined as 28 to 31 completed weeks; moderate to late preterm is defined as 32–36 completed weeks; term is defined as 37–41 completed weeks; and post-term is defined as ≥ 42 weeks. p-values that survive multiple comparison correction (p < 0.001) are marked with an asterisk (*)

Compared to individuals born term, those born extremely preterm (HR = 3.39 [95%CI, 3.13–3.68], very preterm (HR = 2.04 [95%CI, 1.91–2.16], and moderate-late preterm (HR = 1.23, 95%CI, 1.19–1.26) but not post-term (HR = 0.98, 95%CI, 0.95–1.02) had higher risk for any neurodevelopmental or psychiatric disorder (Table 2**)**. When we investigated the individual F-diagnoses separately, statistically significant effect sizes for all gestational age categories but post-term were found for anxiety disorders (HRs ranging from 1.20 to 2.35 across the gestational age categories), intellectual disabilities (HRs ranging from 2.23 to 10.7), SDD (HRs ranging from 1.62 to 8.91), autism spectrum disorders (ASD) (HRs ranging from 1.25 to 4.89), ADHD (HRs ranging from 1.47 to 4.72), and other behavioural and emotional disorders (HRs ranging from 1.48 to 4.69). Extremely PTB was associated also with mood disorders, personality disorders and conduct disorders at statistically significant effect sizes (HRs ranging from 1.98 to 7.50) (Table 2**, **Fig. 2).

**Table 2:** Risk for neurodevelopmental and psychiatric disorders in individuals as a function of gestational age and size for gestational age (All singleton spontaneous delivery births between 1996 and 2014 in Finland followed until 2018, N = 819 764)

	Any F-diagnosis	Psychotic disorders	Mood disorders	Anxiety disorders	Eating disorders	Sleeping disorders	Personality disorders	Intellectual disabilities	SDD	ASD	ADHD	Conduct disorders	Other disorders
Risk for neurodevelopmental and psychiatric disorders													
Exposures													
N	137,163	2227	32,361	42,304	4939	5064	2600	6379	44,720	8719	21,879	3715	22,111
Gestational age													
Term (Reference)	1.00	1.00	1.00	1.00	1.00	1.00	1.00	1.00	1.00	1.00	1.00	1.00	1.00
< 28 weeks	3.39* (3.13–3.68)	1.23 (0.39–3.85)	1.98* (1.58–2.48)	2.35* (1.96–2.81)	2.37 (1.38–4.09)	1.20 (0.62–2.32)	7.50* (4.93–11.43)	10.70* (8.69–13.17)	8.91* (8.18–9.71)	4.89* (3.79–6.31)	4.72^*^ (3.96–5.63)	2.41^*^ (1.33–4.35)	4.69* (3.96–5.56)
28–31 weeks	2.04* (1.91–2.16)	1.40 (0.76–2.55)	1.28 (1.10–1.51)	1.60* (1.42–1.81)	1.76 (1.23–2.49)	1.49 (1.04–2.14)	1.33 (0.77–2.29)	5.37* (4.49–6.42)	3.92* (3.63–4.24)	2.11* (1.68–2.65)	2.39^*^ (2.07–2.76)	1.51 (0.99–2.32)	2.91* (2.56–3.31)
< 32 weeks	2.38* (2.26–2.50)	1.41 (0.83–2.39)	1.45* (1.28–1.66)	1.79* (1.62–1.98)	1.90* (1.41–2.56)	1.42 (1.03–1.94)	2.75* (1.97–3.85)	6.81* (5.94–7.81)	5.25* (4.96–5.57)	2.83* (2.38–3.35)	2.98^*^ (2.66–3.33)	1.73^*^ (1.22–2.46)	3.38* (3.05–3.74)
32–36 weeks	1.23* (1.19–1.26)	0.98 (0.80–1.20)	1.11* (1.05–1.17)	1.20* (1.14–1.25)	1.14 (0.99–1.32)	1.34* (1.18–1.52)	1.44* (1.21–1.71)	2.23* (2.04–2.44)	1.62* (1.57–1.69)	1.25* (1.13–1.37)	1.44^*^ (1.36–1.53)	1.28 (1.10–1.49)	1.48* (1.39–1.57)
≥ 42 weeks	0.98 (0.95–1.02)	0.90 (0.71–1.15)	1.07 (1.01–1.14)	1.01 (0.95–1.06)	0.91 (0.77–1.08)	0.99 (0.84–1.16)	0.91 (0.72–1.15)	1.06 (0.91–1.23)	1.09 (1.03–1.15)	1.05 (0.92–1.18)	1.03 (0.95–1.12)	0.93 (0.76–1.13)	1.02 (0.94–1.10)
Size for gestational age													
AGA (Reference)	1.00	1.00	1.00	1.00	1.00	1.00	1.00	1.00	1.00	1.00	1.00	1.00	1.00
SGA	1.29* (1.26–1.33)	1.17 (0.91–1.49)	1.19* (1.12–1.26)	1.20* (1.14–1.27)	1.26 (1.08–1.47)	1.17 (1.01–1.36)	1.52* (1.26–1.83)	3.62* (3.32–3.94)	1.89* (1.81–1.97)	1.46* (1.32–1.62)	1.49^*^ (1.40–1.59)	1.21 (1.02–1.44)	1.58* (1.49–1.69)
LGA	1.05 (1.02–1.09)	0.84 (0.63–1.11)	1.01 (0.95–1.09)	1.00 (0.94–1.07)	0.87 (0.72–1.05)	1.19 (1.00–1.41)	1.09 (0.86–1.38)	1.14 (0.97–1.33)	1.17* (1.10–1.24)	1.10 (0.96–1.27)	1.03 (0.94–1.13)	1.14 (0.93–1.40)	1.14 (1.05–1.24)
Combined birth outcomes													
Term & AGA (Reference)	1.00	1.00	1.00	1.00	1.00	1.00	1.00	1.00	1.00	1.00	1.00	1.00	1.00
< 32 weeks & SGA	3.16* (2.82–3.54)	0.75 (0.11–5.32)	1.16 (0.79–1.72)	2.05* (1.59–2.63)	2.57 (1.38–4.79)	0.98 (0.37–2.61)	1.55 (0.50–4.82)	9.25* (6.80–12.58)	7.55* (6.61–8.62)	3.05* (1.97–4.73)	3.75^*^ (2.89–4.87)	NA	3.16* (2.82–3.54)
32–36 weeks & SGA	1.48* (1.36–1.60)	0.98 (0.44–2.19)	1.17 (0.97–1.40)	1.38* (1.19–1.60)	1.21 (0.75–1.95)	1.35 (0.89–2.06)	1.34 (0.74–2.42)	4.74* (3.85–5.84)	2.43* (2.18–2.71)	1.65* (1.25–2.18)	1.78^*^ (1.49–2.12)	1.19 (0.71–2.02)	1.48* (1.36–1.60)
< 32 weeks & LGA	2.04* (1.72–2.43)	0.98 (0.14–6.99)	1.33 (0.86–2.06)	1.83* (1.31–2.54)	2.49 (1.04–5.98)	3.31* (1.65–6.62)	2.47 (0.80–7.66)	4.92* (2.91–8.32)	3.80* (3.01–4.80)	2.15 (1.12–4.13)	2.20^*^ (1.42–3.41)	0.61 (0.09–4.35)	2.04* (1.72–2.43)
32–36 weeks & LGA	1.33* (1.20–1.47)	1.23 (0.55–2.75)	1.16 (0.94–1.45)	1.32 (1.11–1.58)	0.93 (0.50–1.73)	1.65 (1.04–2.62)	1.91 (1.06–3.45)	1.82 (1.23–2.69)	1.67* (1.43–1.96)	1.36 (0.92–1.99)	1.51^*^ (1.19–1.91)	1.54 (0.87–2.71)	1.33* (1.20–1.47)

Data are presented as adjusted hazard ratios (HRs) and (95% CI)

Extremely preterm is defined as < 28 weeks; very preterm is defined as 28 to 31 completed weeks; moderate to late preterm is defined as 32 to 36 completed weeks; and post-term is defined as ≥ 42 weeks

Model adjusted for offspring sex, birth year of a child, maternal age at delivery, mother’s country of birth (Finland or other), mother married at birth (yes/no), mother SES, maternal smoking (yes/no), parity (0 or ≥ 1), maternal obesity (yes/no), maternal inpatient and outpatient psychiatric history (yes/no), maternal N05/N06 purchase during pregnancy, and maternal systemic inflammatory disease (yes/no)

p-values that survived multiple comparison correction (p < 0.001) are marked with an asterisk (*)

*Term* individuals born between 37 and 41 completed weeks; *AGA* appropriate for gestational age; *SGA* small for gestational age; *LGA* large for gestational age. *ID* intellectual disabilities; *SDD* specific developmental disorders; *ASD* autism spectrum disorder; *ADHD* attention-deficit/hyperactivity disorder; and *CD* conduct disorder

### Size for gestational age

Compared to individuals born AGA, those born SGA (HR = 1.29 [95%CI, 1.26–1.33]) had a statistically significant higher risk for any neurodevelopmental or psychiatric disorder (Table 2). When we investigated the individual F-diagnoses separately, individuals born with SGA had higher risks for all disorders studied here except for psychotic, eating, sleeping, or conduct disorders, with the largest effect sizes being for intellectual disabilities, SDD, and other emotional and developmental disorders (HRs ranging from 1.58 to 3.62). Conversely, individuals born with LGA had a mildly higher risk for SDD (HR = 1.17 [95%CI, 1.10–1.24]) (Table 2**, **Fig. 2).

### Combined birth outcomes

Within each PTB category, at least 74% of the newborns were AGA (Table S1). Compared to individuals born at term and AGA, being exposed to both preterm and SGA/LGA births was associated with a higher risk for any neurodevelopmental or psychiatric disorder (Table 2**, **Fig. 2). Statistically significant effect size by combined exposures was found for individuals born extremely/very preterm and being SGA (HR = 3.16, 95%CI, 2.82–3.54), and this effect size was larger than that of exposure to extremely/very preterm (HR = 2.38 [95%CI, 2.26–2.50]) or SGA (HR = 1.29 [95%CI, 1.26–1.33]). Concerning individual F-diagnoses, births being both extremely/very preterm and SGA had higher risks for SDD (HR = 7.55 [95%CI, 6.61–8.62]) compared to births being extremely/very preterm or SGA. These two exposures did not modify each other’s effect on SDD on a multiplicative scale (χ^2^_SDD_ = 0.64, p = 0.43). Likewise, individuals born moderate-late preterm and being SGA implied risk for SDD (HR = 2.43 [95%CI, 2.18–2.71]) higher than that of either of the two exposures, again without effect modification (χ^2^ = 0.78, p = 0.38). LGA, on the other hand, showed a protective effect in births being extremely/very preterm such that combined exposures implied a lower risk for SDD (HR = 3.80 [95%CI, 3.01–4.80]) and other emotional and behavioural disorders (F98, HR = 2.04 [95%CI, 1.72–2.43]) compared to births being preterm or LGA. Here, the two exposures did modify each other’s effect on SDD antagonistically (interaction χ^2^_SDD_ = 8.89, p = 0.0029) but there was no effect modification on F98 (χ^2^_F98_ = 2.0, p = 0.16).

The unadjusted HRs for associations between birth outcomes and neurodevelopmental and psychiatric disorders are presented in Table S5.

### Sensitivity analyses

#### Psychotropic medications

In support of the associations between birth outcomes and F-diagnoses, the risk of dispensation of psychotropic medications was, for each individual drug category studied, statistically significantly higher in individuals who were born extremely preterm (HRs ranging from 2.49 to 3.68) and very preterm (HRs ranging from 1.51 to 2.42) and also in individuals who were born SGA (HRs ranging from 1.14 to 1.51), where effect sizes were higher for anxiolytics and sedatives (N05) and psychostimulants (N06B) than for antidepressants (N06A). Further, the risks of dispensation of N05 and N06B were higher in individuals who were exposed to both moderate-late preterm and SGA births compared to those exposed to only one of these two birth outcomes. LGA, however, had no detectable effect on medication, neither alone nor in combination with PTB (Table 3).

**Table 3:** Hazard ratios for individuals’ psychotropic medication purchase in relation to gestational age and size for gestational age (All singleton spontaneous delivery births between 1996 and 2014 in Finland followed until 2018, N = 819 764)

	Any psychotropic medication	Antipsychotics, anxiolytics, hypnotics, and sedatives	Antidepressants	Stimulants
Exposures				
N	41,460	27,552	9599	12,166
Gestational age, weeks				
Term (Reference)	1.00	1.00	1.00	1.00
< 28 weeks	3.11 (2.65–3.65)	2.99 (2.44–3.66)	2.49 (1.70–3.63)	3.68 (2.84–4.78)
28–31 weeks	2.09 (1.87–2.34)	2.20 (1.93–2.51)	1.51 (1.15–1.96)	2.42 (2.01–2.92)
< 32 weeks	2.34 (2.13–2.56)	2.39 (2.14–2.67)	1.73 (1.39–2.15)	2.74 (2.35–3.19)
32–36 weeks	1.29 (1.24–1.36)	1.30 (1.23–1.37)	1.04 (0.93–1.15)	1.43 (1.32–1.54)
≥ 42 weeks	0.95 (0.90–1.01)	0.94 (0.87–1.01)	1.05 (0.93–1.17)	0.94 (0.84–1.05)
Size for gestational age				
AGA (Reference)	1.00	1.00	1.00	1.00
SGA	1.36 (1.29–1.43)	1.34 (1.26–1.43)	1.14 (1.02–1.27)	1.51 (1.39–1.64)
LGA	1.05 (0.99–1.12)	1.05 (0.97–1.13)	1.07 (0.95–1.21)	1.06 (0.94–1.19)
Birth outcomes combined				
Term & AGA (Reference)	1.00	1.00	1.00	1.00
< 32 weeks & SGA	2.66 (2.09–3.37)	2.49 (1.84–3.35)	1.37 (0.71–2.64)	3.74 (2.55–5.50)
32–36 weeks & SGA	1.81 (1.58–2.06)	1.87 (1.59–2.19)	1.01 (0.71–1.45)	1.98 (1.58–2.47)
< 32 weeks & LGA	2.14 (1.58–2.89)	2.13 (1.46–3.10)	1.83 (0.95–3.53)	2.32 (1.37–3.92)
32–36 weeks & LGA	1.24 (1.03–1.50)	1.18 (0.93–1.49)	1.06 (0.71–1.59)	1.50 (1.10–2.05)

Data are presented as adjusted hazard ratios (HRs) and (95% CI)

Extremely preterm is defined as < 28 weeks; very preterm is defined as 28 to 31 completed weeks; moderate-late preterm is defined as 32 to 36 completed weeks; and post-term is defined as ≥ 42 weeks

The analyses were adjusted for offspring sex, birth year, maternal age at delivery, mother’s country of birth (Finland or not), mother married at birth (yes/no), mother SES, maternal smoking (yes/no), parity (0 or ≥ 1), maternal obesity (yes/no), maternal inpatient and outpatient psychiatric history (yes/no), maternal N05/N06 purchase during pregnancy, and maternal systemic inflammatory disease (yes/no)

Psychotropic medications were defined according to the ATC classification system: antipsychotics, anxiolytics, hypnotics and sedatives (ATC groups N05); antidepressants (ATC group N06A); stimulants (ATC group N06B)

*Term* individuals born between 37 and 41 completed weeks; *AGA* appropriate for gestational age; *SGA* small for gestational age; *LGA* large for gestational age

#### Sibling pair analysis

**Fig. 3:**
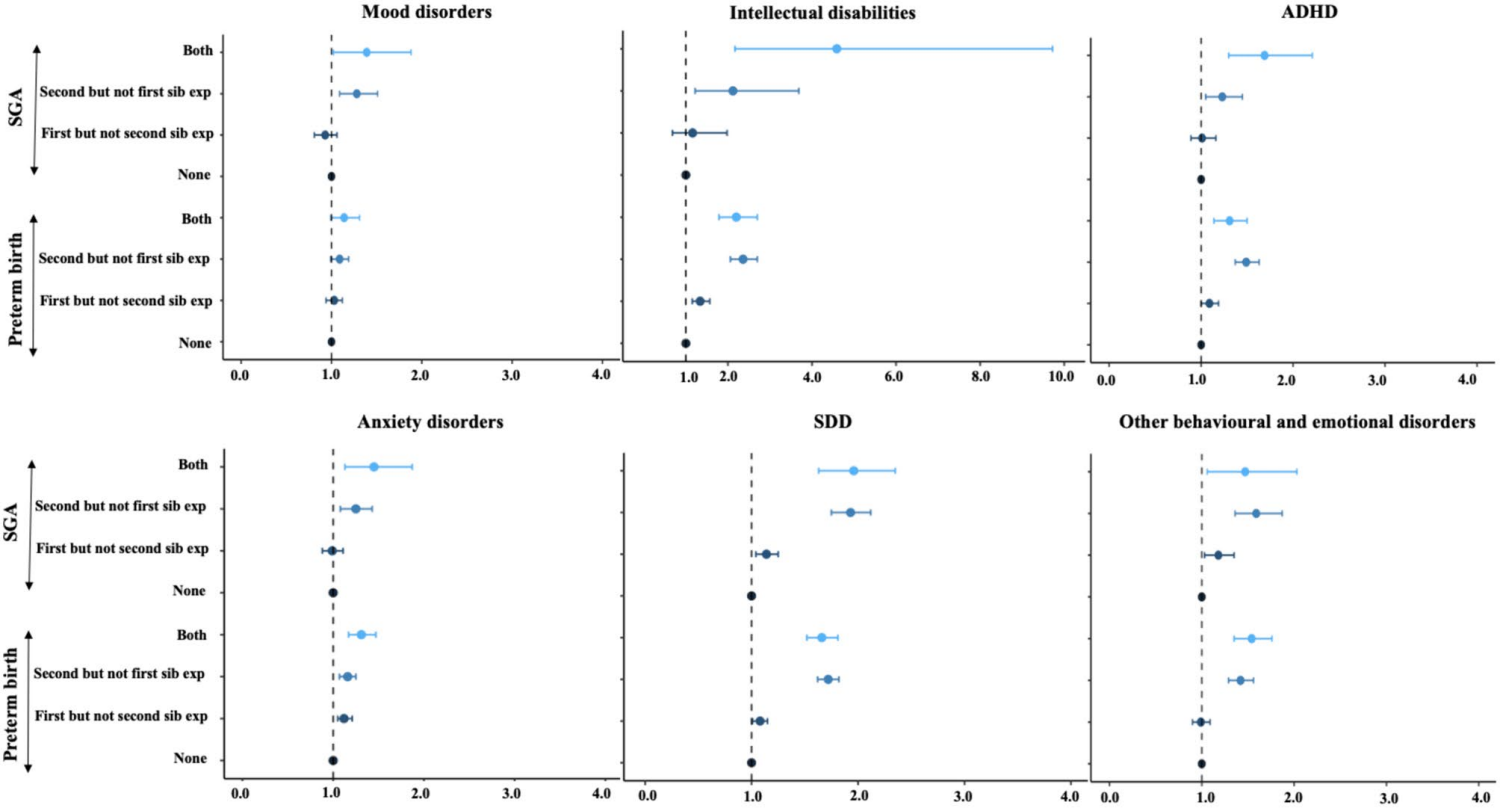
Adjusted hazard ratios (HRs) for diagnosis in the second-born child after exposure to premature birth (< 37 weeks) or small for gestational age (SGA), as estimated by matched sibling pair analysis. Hazard ratios are represented on the x-axis. To better align hazard ratios related to intellectual disabilities, we have plotted them on a separate x-axis range. Abbreviations: both, both siblings in the pair were exposed to preterm birth or SGA; Second but not first sib exp, the second sibling but not the first sibling was exposed to preterm birth or SGA; First but not second sib exp, exposure to the first but not the second sibling; None, none of the siblings in the pair was exposed. The None group was used as a reference

To control for confounding by unmeasured familial risk factors in the associations between birth outcomes and neuropsychiatric disorders, sibling pair analyses were performed focusing on the risk for the second sibling in the pair. Exposure to preterm and SGA births was studied for all F-diagnoses, except psychotic disorders, eating disorders, sleeping disorders, personality disorders, and ASD due to small sample sizes. LGA was not studied due to small main effect sizes. The risk of any neurodevelopmental and psychiatric disorder for the second siblings was higher when these second siblings were exposed to PTB compared to when only the corresponding first siblings were exposed (HR = 1.50 [95%CI, 1.42–1.58] vs HR = 1.07 [95%CI, 1.01–1.13]). This showed that the association between premature birth and any F-diagnosis was not confounded by familial factors. When we investigated the individual F-diagnoses, complete confounding of association was excluded for the PTB associations with intellectual disabilities, SDD, ADHD, and other emotional and developmental disorders (Fig. 3, Table S6).

The association between SGA and any neurodevelopmental and psychiatric disorder was not confounded by familial factors. The risk for the second sibling to develop any F-diagnosis was higher when being exposed to SGA compared to when only first sibling was exposed to SGA (HR = 1.62 [95%CI, 1.48–1.77] vs HR = 1.07 [95%CI, 0.98–1.16]). The individual F-diagnoses associated with SGA without complete familial confounding were mood disorders, SDD, and other emotional and developmental disorders. The effect size was higher also for anxieties, intellectual disabilities and ADHD in second siblings when both siblings were exposed (Fig. 3, Table S7).

#### Sex, age and F98 sub-diagnoses

The effect sizes of sex-stratified associations indicated higher risk among girls than boys for intellectual disabilities, SSD, and other disorders (F98), and higher risk among boys for eating disorders and ASD, by extremely or very PTB (Table S8–S12).

The associations between birth outcomes and anxiety diagnoses remained when restricting age-at-onset to 10 years or older studying those followed at least 10 years, but most associations with mood diagnoses did not remain (Table S13).

In the F98 sub-diagnoses, only the F98.2–F98.3 subgroup showed a statistically significant association with birth outcomes (result S1 of supplementary information, Figure S2).

## Discussion

Using a nationwide cohort of spontaneous births in Finland, we report that PTB across gestational ages and SGA, individually, are associated with higher risks of neurodevelopmental and psychiatric disorders commonly having an early childhood onset, except for sleeping disorders. However, the associations of PTB or SGA with ASD were not assessed for unmeasured shared familial confounding. The effect sizes were higher at lower gestational ages. The largest risks were seen for intellectual disabilities and SDD, with more than five-fold risks for those born extremely or very preterm, compared to term-born peers. Importantly, individuals born preterm being SGA had a higher risk for SDD than those exposed to only one of these birth outcomes. Such elevated combined risk effects were detected for SDD after exposure to extremely/very PTB or moderate-late PTB combined with SGA. Notably, LGA had a protective effect against the risk of extremely/very PTB on SDD and other behavioural and emotional disorders, and LGA and extremely/very PTB modified each other’s effects on SDD antagonistically. In support, PTB and SGA were associated also with a higher dispensation of psychotropic medication. We detected no clear effect on the disorders of post-term birth, or LGA alone. Effect sizes of extremely or very PTB on intellectual disabilities, SDD, and other disorders (F98) were higher in girls than boys, and higher among boys for eating disorders and ASD.

Extremely/very PTB was also associated with personality disorders, mood disorders and anxiety disorders (HRs ranging between 1.98 and 7.50), although familial confounding was not excluded due to smaller sample size. In accordance, extremely/very PTB was associated also with a higher dispensation of anxiolytics and sedatives. SDD, other behavioural and emotional disorders, anxiety, eating, sleeping and personality disorders have rarely been studied as effects of birth outcomes, but a few studies reported a higher risk for mood disorders [32–34]. While effect sizes of PTB combined with SGA on neurodevelopmental disorders are quite unreported, especially for spontaneous births, our findings of the association of PTB or SGA, individually, with risk for intellectual disabilities, ASD and ADHD and conduct disorders, were generally consistent with findings from other cohort-based studies, not stratifying for spontaneous delivery, from Denmark [35], Sweden [6], Norway [36], Finland [11], and USA [37].

Previous population-based studies, not stratifying for mode of delivery, have reported associations between low birth weight (< 2500 g) and ASD (HR = 2.44 [95%CI, 1.99–2.97]) and ADHD (HR = 1.65 [95%CI, 1.40–1.93]) [38]. In a meta-analysis, Gardener et al., found that SGA, but not PTB, was associated with ASD (odds ratio, OR = 1.35 [95%CI, 1.14–1.61]; OR = 1.16 [95%CI, 0.83–1.62], respectively) [39]. In another meta-analysis, Franz et al., found that both very preterm/very low birth weight (OR = 2.25 [95%CI, 1.56–3.26]) and extremely preterm/extremely low birth weight (OR = 4.05 [95%CI, 2.38–6.87]) were associated the risk of ADHD [3]. Deficits in general cognitive abilities were reported for 19-year-olds born extremely preterm [40]. Further, a meta-analysis showed lower intelligence for those born extremely or very preterm compared to full-term peers [41]. In addition, multiple studies have among male, compared to female, children shown a stronger association between individuals born extremely preterm or very preterm and ADHD and ASD diagnosis [24, 42, 43]. Interestingly, we observed stronger association between very PTB and ASD among males, but could not detect any sex-specific association between extremely or very preterm with ADHD, which is consistent with a Swedish cohort showing a similar risk of ADHD among males and females born extremely or moderately preterm [44].

There are proposed mechanisms for associations between birth outcomes and psychiatric morbidity in offspring. In PTB, the cerebral cortex is often underdeveloped, and more susceptible to acute injury and disrupted development during later ages [45, 46]. Less *in-utero* fetal growth in SGA, with common contributors being poor nutrition and placental insufficiency [47], has in human and animal studies been associated with an overrepresentation of altered brain structures [8]. Both SGA and LGA may be a consequence of a metabolically stressed intrauterine environment, with higher plasma levels of insulin, glucose, leptin and inflammatory markers which can influence the placenta and fetus with the potential to influence brain development [48]. However, it is conceivable that LGA may for babies born preterm reflect a more favourable state for postnatal development. Based on our results, we propose that incomplete brain maturation due to prematurity in combination with less *in-utero* fetal growth, contributes to higher risk of certain neurodevelopmental disorders than that of single adverse birth outcome, and that being LGA may compensate developmentally for some risk of psychopathology associated with premature birth. Our results could benefit risk prediction for neurodevelopmental disorders among those born spontaneously preterm, emphasizing that also birth size status should be considered.

### Limitations

First, the follow-up time did not allow detection of all cases with late-onset psychiatric disorders **(**Table 1**)** [49]. Second, in this study, we categorized birth size status solely at the 5th percentile adjusted for gestational age and sex. Third, while this study adjusted for several potential confounders and performed sibling analysis, unknown paternal history and other unmeasured confounders like genetics, feeding patterns or maternal lifestyles, remain potential limitations. Maternal or paternal genotyping data were unavailable to compute polygenic risk scores, which could account for genetic predisposition. Fourth, while we identified all diagnoses of psychiatric disorders over time, comorbidities and changes in diagnoses were not considered in this study. Fifth, due to the smaller sample size of eating disorders, sleeping disorders, ASD and conduct disorder, sibling analysis to investigate familial confounders was not feasible. Lastly, this study is based on the Finnish population; therefore, the generalizability of our findings to non-European populations such as Asian or African populations may be limited.

## Conclusions

Preterm and SGA births were associated with increased risks for childhood-onset neurodevelopmental disorders in individuals with spontaneous births. These two exposures combined implied a higher risk for SDD than one exposure alone, while, being born LGA lowered the risks for SDD and other emotionally or behavioural disorders in individuals born very preterm. Furthermore, notable sex-specific effects were observed in extremely or very PTB, on eating disorders, intellectual disabilities, SDD, ASD and other behavioural and emotional disorders.

## Supplementary Information

Below is the link to the electronic supplementary material.

Supplementary file1 (PDF 909 kb)

## Data Availability

Data are available upon permit from the Social and Health Data Permit Authority Findata (https://www.findata.fi).

## References

[1] Sacchi C, Marino C, Nosarti C, Vieno A, Visentin S, Simonelli A (2020) Association of intrauterine growth restriction and small for gestational age status with childhood cognitive outcomes: a systematic review and meta-analysis. JAMA Pediatr 174:772–781 32453414 10.1001/jamapediatrics.2020.1097 PMC7251506

[2] Saigal S, Doyle LW (2008) An overview of mortality and sequelae of preterm birth from infancy to adulthood. Lancet 371:261–269 18207020 10.1016/S0140-6736(08)60136-1

[3] Franz AP, Bolat GU, Bolat H, Matijasevich A, Santos IS, Silveira RC, Procianoy RS, Rohde LA, Moreira-Maia CR (2018) Attention-deficit/hyperactivity disorder and very preterm/very low birth weight: a meta-analysis. Pediatrics. 10.1542/peds.2017-1645 29255083 10.1542/peds.2017-1645

[4] Johnson S, Hollis C, Kochhar P, Hennessy E, Wolke D, Marlow N (2010) Psychiatric disorders in extremely preterm children: longitudinal finding at age 11 years in the EPICure study. J Am Acad Child Adolesc Psychiatry 49:453-463.e451 20431465

[5] Johnson S, Marlow N (2011) Preterm birth and childhood psychiatric disorders. Pediatr Res 69:11–18 10.1203/PDR.0b013e318212faa0 21289534

[6] Lindström K, Lindblad F, Hjern A (2011) Preterm birth and attention-deficit/hyperactivity disorder in schoolchildren. Pediatrics 127:858–865 21502231 10.1542/peds.2010-1279

[7] Kerr-Wilson CO, Mackay DF, Smith GCS, Pell JP (2011) Meta-analysis of the association between preterm delivery and intelligence. J Public Health 34:209–216 10.1093/pubmed/fdr024 21393308

[8] de Bie HM, Oostrom KJ, Delemarre-van de Waal HA (2010) Brain development, intelligence and cognitive outcome in children born small for gestational age. Horm Res Paediatr 73:6–14 20190535 10.1159/000271911

[9] Jenabi E, Bashirian S, Asali Z, Seyedi M (2021) Association between small for gestational age and risk of autism spectrum disorders: a meta-analysis. Clin Exp Pediatr 64:538–542 33539699 10.3345/cep.2020.01956 PMC8498018

[10] Heinonen K, Räikkönen K, Pesonen AK, Andersson S, Kajantie E, Eriksson JG, Wolke D, Lano A (2010) Behavioural symptoms of attention deficit/hyperactivity disorder in preterm and term children born small and appropriate for gestational age: a longitudinal study. BMC Pediatr 10:91 21159164 10.1186/1471-2431-10-91 PMC3012665

[11] Sucksdorff M, Lehtonen L, Chudal R, Suominen A, Joelsson P, Gissler M, Sourander A (2015) Preterm birth and poor fetal growth as risk factors of attention-deficit/ hyperactivity disorder. Pediatrics 136:e599-608 26304830 10.1542/peds.2015-1043

[12] Persson M, Opdahl S, Risnes K, Gross R, Kajantie E, Reichenberg A, Gissler M, Sandin S (2020) Gestational age and the risk of autism spectrum disorder in Sweden, Finland, and Norway: a cohort study. PLoS Med 17:e1003207 32960896 10.1371/journal.pmed.1003207 PMC7508401

[13] Boog G (2004) Obstetrical complications and subsequent schizophrenia in adolescent and young adult offsprings: is there a relationship? Eur J Obstet Gynecol Reprod Biol 114:130–136 15140504 10.1016/j.ejogrb.2003.09.041

[14] Aarnoudse-Moens CS, Weisglas-Kuperus N, van Goudoever JB, Oosterlaan J (2009) Meta-analysis of neurobehavioral outcomes in very preterm and/or very low birth weight children. Pediatrics 124:717–728 19651588 10.1542/peds.2008-2816

[15] Hutcheon JA, Zhang X, Platt RW, Cnattingius S, Kramer MS (2011) The case against customised birthweight standards. Paediatr Perinat Epidemiol 25:11–16 21133965 10.1111/j.1365-3016.2010.01155.x

[16] Lawn JE, Ohuma EO, Bradley E, Idueta LS, Hazel E, Okwaraji YB, Erchick DJ, Yargawa J, Katz J, Lee AC (2023) Small babies, big risks: global estimates of prevalence and mortality for vulnerable newborns to accelerate change and improve counting. Lancet 401:1707–1719 37167989 10.1016/S0140-6736(23)00522-6

[17] Zhang T, Sidorchuk A, Sevilla-Cermeño L, Vilaplana-Pérez A, Chang Z, Larsson H, Mataix-Cols D, de la Cruz LF (2019) Association of cesarean delivery with risk of neurodevelopmental and psychiatric disorders in the offspring: a systematic review and meta-analysis. JAMA Netw Open 2:e1910236–e1910236 31461150 10.1001/jamanetworkopen.2019.10236 PMC6716295

[18] Strang JF, Kenworthy L, Dominska A, Sokoloff J, Kenealy LE, Berl M, Walsh K, Menvielle E, Slesaransky-Poe G, Kim K-E (2014) Increased gender variance in autism spectrum disorders and attention deficit hyperactivity disorder. Arch Sex Behav 43:1525–1533 24619651 10.1007/s10508-014-0285-3

[19] Van Lieshout RJ, Boyle MH (2011) Is bigger better? Macrosomia and psychopathology later in life. Obes Rev 12:e405–e411 20977604 10.1111/j.1467-789X.2010.00816.x

[20] Van Lieshout RJ, Savoy CD, Ferro MA, Krzeczkowski JE, Colman I (2020) Macrosomia and psychiatric risk in adolescence. Eur Child Adolesc Psychiatry 29:1537–1545 31894421 10.1007/s00787-019-01466-7

[21] Strang JF, Kenworthy L, Dominska A, Sokoloff J, Kenealy LE, Berl M, Walsh K, Menvielle E, Slesaransky-Poe G, Kim K-E, Luong-Tran C, Meagher H, Wallace GL (2014) Increased gender variance in autism spectrum disorders and attention deficit hyperactivity disorder. Arch Sex Behav 43:1525–1533 24619651 10.1007/s10508-014-0285-3

[22] Davies W (2014) Sex differences in attention deficit hyperactivity disorder: candidate genetic and endocrine mechanisms. Front Neuroendocrinol 35:331–346 24680800 10.1016/j.yfrne.2014.03.003

[23] Allen L, Leon-Attia O, Shaham M, Shefer S, Gabis LV (2020) Autism risk linked to prematurity is more accentuated in girls. PLoS ONE 15:e0236994 32854110 10.1371/journal.pone.0236994 PMC7452728

[24] Bröring T, Oostrom KJ, van Dijk-Lokkart EM, Lafeber HN, Brugman A, Oosterlaan J (2018) Attention deficit hyperactivity disorder and autism spectrum disorder symptoms in school-age children born very preterm. Res Dev Disabil 74:103–112 29413425 10.1016/j.ridd.2018.01.001

[25] Hintz SR, Kendrick DE, Vohr BR, Kenneth Poole W, Higgins RD (2006) Gender differences in neurodevelopmental outcomes among extremely preterm, extremely-low-birthweight infants. Acta Paediatr 95:1239–1248 16982497 10.1080/08035250600599727

[26] Leppert B, Havdahl A, Riglin L, Jones HJ, Zheng J, Davey Smith G, Tilling K, Thapar A, Reichborn-Kjennerud T, Stergiakouli E (2019) Association of maternal neurodevelopmental risk alleles with early-life exposures. JAMA Psychiat 76:834–842 10.1001/jamapsychiatry.2019.0774 PMC6495368 31042271

[27] Havdahl A, Wootton RE, Leppert B, Riglin L, Ask H, Tesli M, Bugge Askeland R, Hannigan LJ, Corfield E, Øyen A-S, Andreassen OA, Tilling K, Davey Smith G, Thapar A, Reichborn-Kjennerud T, Stergiakouli E (2022) Associations between pregnancy-related predisposing factors for offspring neurodevelopmental conditions and parental genetic liability to attention-deficit/hyperactivity disorder, autism, and schizophrenia: the Norwegian mother, father and child cohort study (MoBa). JAMA Psychiat 79:799–810 10.1001/jamapsychiatry.2022.1728 PMC9260642 35793100

[28] Skoglund C, Chen Q, D’Onofrio BM, Lichtenstein P, Larsson H (2014) Familial confounding of the association between maternal smoking during pregnancy and ADHD in offspring. J Child Psychol Psychiatr 55:61–68 10.1111/jcpp.12124 PMC4217138 25359172

[29] Sankilampi U, Hannila ML, Saari A, Gissler M, Dunkel L (2013) New population-based references for birth weight, length, and head circumference in singletons and twins from 23 to 43 gestation weeks. Ann Med 45:446–454 23768051 10.3109/07853890.2013.803739

[30] Clayton PE, Cianfarani S, Czernichow P, Johannsson G, Rapaport R, Rogol A (2007) Management of the child born small for gestational age through to adulthood: a consensus statement of the international societies of pediatric endocrinology and the growth hormone research society. J Clin Endocrinol Metab 92:804–810 17200164 10.1210/jc.2006-2017

[31] Lampi KM, Sourander A, Gissler M, Niemelä S, Rehnström K, Pulkkinen E, Peltonen L, Von Wendt L (2010) Brief report: validity of Finnish registry-based diagnoses of autism with the ADI-R. Acta Paediatr 99:1425–1428 20412100 10.1111/j.1651-2227.2010.01835.x

[32] Nosarti C, Reichenberg A, Murray RM, Cnattingius S, Lambe MP, Yin L, MacCabe J, Rifkin L, Hultman CM (2012) Preterm birth and psychiatric disorders in young adult life. Arch Gen Psychiatry 69:610–617 10.1001/archgenpsychiatry.2011.1374 22660967

[33] Lindström K, Lindblad F, Hjern A (2009) Psychiatric morbidity in adolescents and young adults born preterm: a Swedish national cohort study. Pediatrics 123:e47–e53 19117846 10.1542/peds.2008-1654

[34] Pettersson E, Larsson H, D’Onofrio B, Almqvist C, Lichtenstein P (2019) Association of fetal growth with general and specific mental health conditions. JAMA Psychiat 76:536–543 10.1001/jamapsychiatry.2018.4342 PMC6495458 30725083

[35] Atladottir H, Schendel D, Henriksen T, Hjort L, Parner E (2016) Gestational age and autism spectrum disorder: trends in risk over time. Autism Res 9:224–231 26363410 10.1002/aur.1525

[36] Ask H, Gustavson K, Ystrom E, Havdahl KA, Tesli M, Askeland RB, Reichborn-Kjennerud T (2018) Association of gestational age at birth with symptoms of attention-deficit/hyperactivity disorder in children. JAMA Pediatr 172:749–756 29946656 10.1001/jamapediatrics.2018.1315 PMC6142916

[37] Singh GK, Kenney MK, Ghandour RM, Kogan MD, Lu MC (2013) Mental health outcomes in US children and adolescents born prematurely or with low birthweight. Depress Res Treat 2013:1–13 10.1155/2013/570743 PMC3845867 24324882

[38] Class QA, Rickert ME, Larsson H, Lichtenstein P, D’Onofrio BM (2014) Fetal growth and psychiatric and socioeconomic problems: population-based sibling comparison. Br J Psychiatry 205:355–361 25257067 10.1192/bjp.bp.113.143693 PMC4217026

[39] Gardener H, Spiegelman D, Buka SL (2011) Perinatal and neonatal risk factors for autism: a comprehensive meta-analysis. Pediatrics 128:344–355 21746727 10.1542/peds.2010-1036 PMC3387855

[40] O’Reilly H, Johnson S, Ni Y, Wolke D, Marlow N (2020) Neuropsychological outcomes at 19 years of age following extremely preterm birth. Pediatrics 145:e20192087 31924688 10.1542/peds.2019-2087

[41] Twilhaar ES, Wade RM, de Kieviet JF, van Goudoever JB, van Elburg RM, Oosterlaan J (2018) Cognitive outcomes of children born extremely or very preterm since the 1990s and associated risk factors: a meta-analysis and meta-regression. JAMA Pediatr 172:361–367 29459939 10.1001/jamapediatrics.2017.5323 PMC5875339

[42] Crump C, Sundquist J, Sundquist K (2021) Preterm or early term birth and risk of autism. Pediatrics 148:e2020032300 34380775 10.1542/peds.2020-032300 PMC9809198

[43] Hack M, Taylor HG, Schluchter M, Andreias L, Drotar D, Klein N (2009) Behavioral outcomes of extremely low birth weight children at age 8 years. J Dev Behav Pediatr 30:122–130 19322106 10.1097/DBP.0b013e31819e6a16 PMC3074440

[44] Crump C, Sundquist J, Sundquist K (2023) Preterm or early term birth and risk of attention-deficit/hyperactivity disorder: a national cohort and co-sibling study. Ann Epidemiol 86:119-125.e114 37648179 10.1016/j.annepidem.2023.08.007 PMC10538375

[45] Nivins S, Kennedy E, McKinlay C, Thompson B, Harding JE, Alsweiler J, Brown G, Gamble G, Wouldes T, Keegan P, Harris D, Chase G, Turuwhenua J, Rogers J, Shah R, Dai D, Ledger J, Macdonald S, McNeill A, Bevan C, Burakevych N, May R, Hossin S, McKnight G, Hasan R, Wilson J, Knopp J, Chakraborty A, Zhou T, Miller S, Children with H, Their Later Development Study T, Steering g, Other members of the CM-cOSt (2023) Size at birth predicts later brain volumes. Sci Rep 13:12446 37528153 10.1038/s41598-023-39663-9 PMC10393952

[46] Volpe JJ (2009) Brain injury in premature infants: a complex amalgam of destructive and developmental disturbances. Lancet Neurol 8:110–124 19081519 10.1016/S1474-4422(08)70294-1 PMC2707149

[47] Sharma D, Shastri S, Sharma P (2016) Intrauterine growth restriction: antenatal and postnatal aspects. Clin Med Insights Pediatr 10:67–83 27441006 10.4137/CMPed.S40070 PMC4946587

[48] Kong L, Chen X, Gissler M, Lavebratt C (2020) Relationship of prenatal maternal obesity and diabetes to offspring neurodevelopmental and psychiatric disorders: a narrative review. Int J Obes (Lond) 44:1981–2000 32494038 10.1038/s41366-020-0609-4 PMC7508672

[49] Solmi M, Radua J, Olivola M, Croce E, Soardo L, Salazar de Pablo G, Il Shin J, Kirkbride JB, Jones P, Kim JH (2022) Age at onset of mental disorders worldwide: large-scale meta-analysis of 192 epidemiological studies. Mol Psychiatry 27:281–295 34079068 10.1038/s41380-021-01161-7 PMC8960395

