# Supplementary material for: Association of preterm birth and birth size status with neurodevelopmental and psychiatric disorders in spontaneous births
Source: Eur Child Adolesc Psychiatry. 2024 Jun 12;34(1):261–73. doi: 10.1007/s00787-024-02489-5 (PMC11805797; doi:10.1007/s00787-024-02489-5)
Supplement: Supplementary file 1 — Supplementary file1 (PDF 909 kb) [file 787_2024_2489_MOESM1_ESM.pdf]

Supplementary Information

Association of preterm birth and birth size status with neurodevelopmental and psychiatric disorders in spontaneous births

European Child and Adolescent Psychiatry

Linghua Kong PhD [a,b,c*], Samson Nivins, PhD [d*], Xinxia Chen PhD [a,b,c], Yajun Liang PhD [e], Mika Gissler PhD [b,c,f], Catharina Lavebratt MSc, PhD [b,c,†]

* contributed equally as co-first authors

[a] School of Nursing and Rehabilitation, Cheeloo College of Medicine, Shandong University, Shandong, China
[b] Department of Molecular Medicine and Surgery, Karolinska Institutet, Stockholm, Sweden
[c] Center for Molecular Medicine, Karolinska University Hospital, Stockholm, Sweden
[d] Department of Neuroscience, Karolinska Institutet, Stockholm, Sweden
[e] Department of Global Public Health, Karolinska Institutet, Stockholm, Sweden
[f] Department of Knowledge Brokers, Finnish Institute for Health and Welfare, Helsinki, Finland

†*Corresponding author:* Catharina Lavebratt
Translational Psychiatry Unit, Centre for Molecular Medicine, Karolinska University Hospital L8:00, 171 76 Stockholm, Sweden. Phone: +46-8-51776524; Fax: +46-8-51773909; E-mail: catharina.lavebratt@ki.se

Table of contents

Results S1. Subgroup analysis for children with F98 diagnosis

Table S1. Characteristics of all singleton spontaneous delivery births between 1996 and 2014 in Finland stratified by gestational age and size for gestational age (N=819 764)

Table S2. Numbers of individuals with psychiatric diagnosis and psychotropic medication use stratified by gestational age and size for gestational age (All live singleton spontaneous delivery births between 1996 and 2014 in Finland followed until 2018 (N=819 764)

Table S3. Numbers of individuals with psychiatric diagnosis and psychotropic medication use stratified by gestational age or size for gestational age (All live singleton spontaneous delivery births between 1996 and 2014 in Finland followed until 2018 (N=819 764)

Table S4. Numbers of individuals with psychiatric diagnosis stratified by sex, gestational age, and size for gestational age (All live singleton spontaneous delivery births between 1996 and 2014 in Finland followed until 2018 (N=819 764)

Table S5. Crude hazard ratios for neurodevelopmental and psychiatric disorders in individuals as a function of gestational age and size for gestational age (All live singleton spontaneous delivery births between 1996 and 2014 in Finland followed until 2018, N=819 764)

Table S6. Sibling analysis: Risk for neurodevelopmental and psychiatric disorders in second-born child after exposure to premature birth (before 37 gestational weeks), as estimated by matched sibling pair analysis. All singleton sibling pairs (n=299 331) among the 819 764 births (born 1996-2014) were included and followed-up until 2018

Table S7. Sibling analysis: Risk for neurodevelopmental and psychiatric disorders in second-born child after the exposure being born small for gestational age (SGA), as estimated by sibling pair analysis. All singleton sibling pairs (n=299 331) among the 819 764 births (born 1996-2014) were included and followed-up until 2018

Table S8. Boys: Adjusted hazard ratios (HRs) for neurodevelopmental and psychiatric disorders in boys in relation to gestational age and size for gestational age (All live singleton spontaneous delivery births between 1996 and 2014 in Finland followed until 2018 (N=418 335)

Table S9. Girls: Adjusted hazard ratios (HRs) for neurodevelopmental and psychiatric disorders in girls in relation to gestational age and size for gestational age (All live singleton spontaneous delivery births between 1996 and 2014 in Finland followed until 2018 (N=401 429)

Table S10. Numbers of individuals with psychotropic medication use stratified by sex, gestational age, and size for gestational age (All live singleton spontaneous delivery births between 1996 and 2014 in Finland followed until 2018, N=819 764)

Table S11. Hazard ratios for boys' psychotropic medication use in relation to gestational age and size for gestational age (All live singleton spontaneous delivery births between 1996 and 2014 in Finland followed until 2018)

Table S12. Hazard ratios for girls' psychotropic medication use in relation to gestational age and size for gestational age (All live singleton spontaneous delivery births between 1996 and 2014 in Finland followed until 2018)

Table S13. Adjusted hazard ratios (HRs) for mood and anxiety disorders in individuals at least 10 years of age in relation to gestational age and size for gestational age (N=561 350, born 1996-2008 followed until 2018)

Fig S1. Directed acyclic graph representing the causal assumptions used for covariate selection based on existing literature, where nodes represent ancestors of exposures and/or outcomes, and arrows represent biasing or causal paths.

Fig S2. Adjusted hazard ratios (HRs) for feeding or eating disorders of childhood (F98.2-3), in relation to gestational age and size for gestational age

Results S1. Subgroup analysis for children with F98 diagnosis

Since F98 includes a range of disorders and was associated with birth outcomes, we carried out an F98 subgroup analysis, grouping as follows: F98.0-F98.1, F98.2-F98.3, F98.4, F98.5, F98.8 and F98.9. Only F98.0-F98.1 and F98.2-F98.3 had adequate sample size and were studied further. Only the F98.2-3 subgroup showed a significant association with birth outcomes at HRs in the range of 1.25 to 2.05 **(eFigure 2)**.

Table S1. Characteristics of all singleton spontaneous delivery births between 1996 and 2014 in Finland stratified by gestational age and size for gestational age (N=819 764)

Variable	Extremely preterm	Very preterm	Moderate-late preterm	Term	Post-term	SGA	AGA	LGA
N	1392	3334	30533	759498	21977	22969	775507	18258
**Birth year of a child**								
1996-1999	277 (19.9)	722 (21.7) *	6649 (21.8) *	160211 (21.1)	5166 (23.5) *	4733 (20.6)	163540 (21.1)	4752 (26.0) *
2000-2004	351 (25.2)	942 (28.2)	8189 (26.8)	194684 (25.6)	6412 (29.2)	5906 (25.7)	199495 (25.7)	5177 (28.3)
2005-2009	367 (26.4)	826 (24.8)	7997 (26.2)	204648 (26.9)	6175 (28.1)	6435 (28.0)	209233 (27.0)	4345 (23.8)
2010-2014	397 (28.5)	844 (25.3)	7698 (25.2)	199955 (26.3)	4224 (19.2)	5895 (25.7)	203239 (26.2)	3984 (21.8)
**Sex of a child**								
Boy	738 (53.0)	1829 (54.9) *	17293 (56.6) *	385885 (50.8)	11064 (50.3)	11898 (51.8)	395901 (51.1)	9010 (49.4) *
Girl	654 (47.0)	1505 (45.1)	13240 (43.4)	373613 (49.2)	10913 (49.7)	11071 (48.2)	379606 (48.9)	9248 (50.6)
**Size for gestational age**								
SGA	168 (12.1) *	558 (16.7) *	2448 (8.0) *	19033 (2.5)	430 (2.0) *	22969 (100)	0 (0)	0 (0)
AGA	1092 (78.5)	2480 (74.4)	26039 (85.3)	717973 (94.5)	20858 (94.9)	0 (0)	775507 (100)	0 (0)
LGA	116 (8.3)	245 (7.4)	1624 (5.3)	15425 (2.0)	337 (1.5)	0 (0)	0 (0)	18258 (100)
**Maternal data**								
**Maternal age (years)**								
< 20	45 (3.2) *	97 (2.9) *	1039 (3.4) *	20355 (2.7)	735 (3.3) *	973 (4.2) *	21041 (2.7)	257 (1.4) *
20 – 24	199 (14.3)	527 (15.8)	5108 (16.7)	128190 (16.9)	3936 (17.9)	4450 (19.4)	131288 (16.9)	2222 (12.2)
25 – 29	377 (27.1)	933 (28.0)	9380 (30.7)	249517 (32.8)	7190 (32.7)	6959 (30.3)	255026 (32.9)	5412 (29.6)
30 – 34	407 (29.2)	967 (29.0)	8952 (29.3)	234636 (30.9)	6587 (30.0)	6413 (27.9)	238912 (30.8)	6224 (34.1)
⩾ 35	364 (26.2)	810 (24.3)	6054 (19.8)	126799 (16.7)	3529 (16.1)	4174 (18.2)	129239 (16.7)	4143 (22.7)
**Parity**								
0	666 (47.8) *	1712 (51.4) *	15573 (51.0) *	308511 (40.6)	11735 (53.4) *	13934 (60.7) *	320202 (41.3)	4061 (22.2) *
⩾ 1	725 (52.1)	1616 (48.5)	14938 (48.9)	450682 (59.3)	10228 (46.5)	9016 (39.2)	454982 (58.7)	14191 (77.7)
Missing	1 (0.07)	6 (0.18)	22 (0.07)	305 (0.04)	14 (0.06)	19 (0.08)	323 (0.04)	6 (0.03)
**Socioeconomic status (SES)**								

Upper white collar	196 (14.1) *	499 (15.0) *	4714 (15.4) *	127723 (16.8)	3836 (17.4) *	3257 (14.2) *	130730 (16.9)	2981 (16.3) *
Lower white collar	465 (33.4)	1166 (35.0)	10876 (35.6)	267888 (35.3)	7385 (33.6)	7663 (33.4)	273190 (35.2)	6927 (37.9)
Blue collar	227 (16.3)	517 (15.5)	4712 (15.4)	109228 (14.4)	3219 (14.6)	3894 (16.9)	111201 (14.3)	2808 (15.4)
Others	254 (18.2)	578 (17.3)	5448 (17.8)	134722 (17.7)	4022 (18.3)	4198 (18.3)	137641 (17.8)	3185 (17.4)
Missing	250 (18.0)	574 (17.2)	4783 (15.7)	119937 (15.8)	3515 (16.0)	3957 (17.2)	122745 (15.8)	2357 (12.9)
**Marital status**								
Married	770 (55.3)	1813 (54.4) *	16997 (55.7) *	451363 (59.4)	12169 (55.4) *	11544 (50.3) *	459543 (59.3)	12025 (65.9) *
Cohabiting	414 (29.7)	985 (29.5)	9217 (30.2)	220706 (29.1)	6696 (30.5)	7720 (33.6)	225910 (29.1)	4388 (24.0)
Other	172 (12.4)	446 (13.4)	3618 (11.8)	73280 (9.6)	2622 (11.9)	3166 (13.8)	75492 (9.7)	1480 (8.1)
Missing	36 (2.6)	90 (2.7)	701 (2.3)	14149 (1.9)	490 (2.2)	539 (2.3)	14562 (1.9)	365 (2.0)
**Country of birth**								
Finland	1214 (87.2)	3057 (91.7)	28024 (91.8)	697861 (91.9)	19951 (90.8)	20580 (89.6) *	712501 (91.9)	17026 (93.2)
Other	178 (12.8)	277 (8.3)	2509 (8.2)	61637 (8.1)	2026 (9.2)	2389 (10.4)	63006 (8.1)	1232 (6.8)
**Smoking**								
No	1023 (73.5)*	2472 (74.1) *	24128 (79.0) *	629907 (82.9)	18045 (82.1) *	16247 (70.7) *	643203 (82.9)	16125 (88.3) *
Stopped during first trimester	39 (2.8)	110 (3.3)	1091 (3.6)	27632 (3.6)	984 (4.48)	846 (3.7)	28445 (3.7)	565 (3.1)
Continued	233 (16.7)	533 (16.0)	4259 (13.9)	84360 (11.1)	2515 (11.44)	5285 (23.0)	85546 (11.0)	1069 (5.8)
Missing	97 (7.0)	219 (6.6)	1055 (3.5)	17599 (2.3)	433 (1.97)	591 (2.6)	18313 (2.4)	499 (2.8)
**Psychiatric history – inpatient care (1987-2014)**								
Yes	49 (3.5) *	108 (3.2) *	883 (2.9) *	15689 (2.1)	462 (2.1)	755 (3.3) *	16080 (2.1)	356 (2.0)
No	1343 (96.5)	3226 (96.8)	29650 (97.1)	743809 (97.9)	21515 (97.9)	22214 (96.7)	759427 (97.9)	17902 (98.0)
**Psychiatric history – outpatient care (1998-2014)**								
Yes	179 (12.9) *	321 (9.6) *	2662 (8.7) *	50548 (6.7)	1334 (6.1) *	2109 (9.2) *	51933 (6.7)	1002 (5.5) *
No	1213 (87.1)	3013 (90.4)	27871 (91.3)	708950 (93.3)	20643 (93.9)	20860 (90.8)	723574 (93.3)	17256 (94.5)
**Maternal systemic inflammatory disease**								
Yes	27 (1.9) *	51 (1.5)	472 (1.5) *	7368 (1.0)	183 (0.8)	319 (1.4) *	7604 (1.0)	178 (1.0)
No	1365 (98.1)	3283 (98.5)	30061 (98.5)	752130 (99.0)	21794 (99.2)	22650 (98.6)	767903 (99.0)	18080 (99.0)
**Maternal use of psychotropic medication during pregnancy**								

Yes	84 (6.0) *	195 (5.8) *	1628 (5.3) *	30924 (4.1)	819 (3.7)	1182 (5.1) *	31697 (4.1)	771 (4.2)
No	1308 (94.0)	3139 (94.2)	28905 (94.7)	728574 (95.9)	21158 (96.3)	21787 (94.9)	743810 (95.9)	17487 (95.8)
**Maternal obesity (ICD 10: E65-66)**								
Yes	56 (4.0) *	131 (3.9) *	904 (3.0) *	16393 (2.2)	600 (2.7) *	443 (1.9)	16784 (2.2)	857 (4.7) *
No	1336 (96.0)	3203 (96.1)	29629 (97.0)	743105 (97.8)	21377 (97.3)	22526 (98.1)	758723 (97.8)	17401 (95.3)

Data are presented as n (%) unless otherwise indicated. Abbreviations: SGA, small for gestational age; AGA, appropriate for gestational age; LGA, large for gestational age; SES, socioeconomic status; and PGDM, pre-gestational diabetes mellitus. LGA and SGA are defined as birth weight and/or birth length, above or below the 2 SDs from the mean for gestation and sex-specific in the Finnish population[1], based on the International Societies of Pediatric Endocrinology and the Growth Hormone Research Society[2]. Extremely preterm is defined as < 28 weeks; very preterm is defined as 28 to 31 completed weeks; moderate-late preterm is defined as 32 to 36 completed weeks; term is defined as 37 to 41 completed weeks; and post-term is defined as ≥42 weeks.

A value of $p < 0.001$ is considered statistically significant and is indicated by an asterisk (*).

Table S2. Numbers of individuals with psychiatric diagnosis and psychotropic medication use stratified by gestational age and size for gestational age (All live singleton spontaneous delivery births between 1996 and 2014 in Finland followed until 2018 (N=819 764)

Variables	Extremely preterm	Very preterm	Preterm (< 32 weeks)	Moderate-late preterm	Full-term	Post-term	SGA	AGA	LGA	Missing
N	1392	3334	4726	30533	759498	21977	22969	775507	18258	3030
**Psychiatric diagnoses (ICD-10)**										
Any F-diagnosis (F00-F99)	572 (41.09)	1021 (30.62)	1593 (33.70)	6249 (20.47)	124779 (16.43)	3924 (17.86)	4908 (21.37)	128206 (16.53)	3431 (18.79)	618 (20.40)
Psychotic disorders (F20-F29)	3 (0.22)	11 (0.33)	14 (0.29)	104 (0.34)	2029 (0.27)	68 (0.31)	66 (0.29)	2099 (0.27)	50 (0.27)	12 (0.40)
Mood disorders (F30-39, F92)	75 (5.39)	154 (4.62)	229 (4.84)	1330 (4.36)	29616 (3.90)	1038 (4.72)	1123 (4.89)	30308 (3.91)	782 (4.28)	148 (4.88)
Anxiety disorders (F40-43, F93)	120 (8.62)	253 (7.59)	373 (7.89)	1886 (6.18)	38583 (5.08)	1261 (5.74)	1486 (6.47)	39617 (5.11)	1000 (5.48)	201 (6.63)
Eating disorders (F50)	13 (0.93)	31 (0.93)	44 (0.93)	197 (0.65)	4541 (0.60)	137 (0.62)	164 (0.71)	4643 (0.60)	112 (0.61)	20 (0.66)
Sleeping disorders (F51)	9 (0.65)	30 (0.90)	39 (0.83)	261 (0.85)	4608 (0.61)	141 (0.64)	176 (0.77)	4740 (0.61)	133 (0.73)	15 (0.50)
Personality disorders (F60-69)	22 (1.58)	13 (0.39)	35 (0.74)	136 (0.45)	2343 (0.31)	72 (0.33)	116 (0.51)	2401 (0.31)	69 (0.38)	14 (0.46)
Intellectual disabilities (F70-79)	91 (6.54)	123 (3.69)	214 (4.52)	504 (1.65)	5452 (0.72)	173 (0.79)	598 (2.60)	5584 (0.72)	161 (0.88)	36 (1.19)
Specific developmental disorders (F80-83)	525 (37.72)	634 (19.02)	1159 (24.52)	2670 (8.74)	39407 (5.19)	1288 (5.86)	2368 (10.31)	41028 (5.29)	1128 (6.18)	196 (6.47)
Autism spectrum disorders (F84)	60 (4.31)	75 (2.25)	135 (2.85)	444 (1.45)	7828 (1.03)	266 (1.21)	390 (1.70)	8082 (1.04)	201 (1.10)	46 (1.52)

ADHD (F90)	126 (9.05)	190 (5.70)	316 (6.69)	1171 (3.84)	19658 (2.59)	642 (2.92)	1002 (4.36)	20321 (2.62)	464 (2.54)	92 (3.04)
Conduct disorders (F91)	11 (0.79)	21 (0.63)	32 (0.68)	181 (0.59)	3385 (0.45)	102 (0.46)	138 (0.60)	3470 (0.45)	92 (0.50)	15 (0.50)
Other emotional & behavioural disorders (F98)	134 (9.63)	237 (7.11)	371 (7.85)	1218 (3.99)	19813 (2.61)	624 (2.84)	993 (4.32)	20492 (2.64)	541 (2.96)	85 (2.81)
Feeding/eating disorders (F98.2-F98.3)	11 (0.79)	23 (0.68)	34 (0.72)	192 (0.62)	3805 (0.50)	98 (0.44)	151 (0.65)	3870 (0.49)	108 (0.59)	17 (0.56)
**Psychotropic medication (ATC)**										
Any medication (N05-N06)	149 (10.70)	316 (9.48)	465 (9.83)	2027 (6.64)	37641 (4.96)	1156 (5.26)	1587 (6.91)	38665 (4.99)	1037 (5.68)	171 (5.64)
Antipsychotics, anxiolytics, hypnotics and sedatives (N05)	94 (6.75)	219 (6.57)	313 (6.62)	1332 (4.36)	25044 (3.30)	755 (3.44)	1026 (4.47)	25721 (3.32)	697 (3.82)	108 (3.56)
Antidepressants (N06A)	27 (1.94)	55 (1.65)	82 (1.73)	368 (1.21)	8803 (1.16)	303 (1.38)	311 (1.35)	8974 (1.16)	271 (1.48)	43 (1.42)
Stimulants (N06B)	57 (4.09)	112 (3.36)	169 (3.57)	696 (2.28)	11020 (1.45)	335 (1.52)	563 (2.45)	11377 (1.47)	280 (1.53)	54 (1.78)

Data are presented as No. (%). Abbreviations: SGA, small for gestational age; AGA, appropriate for gestational age; LGA, large for gestational age, SDD, specific developmental disorders; ASD, autism spectrum disorder; and ADHD, attention-deficit/hyperactivity disorder.

Extremely preterm is defined as < 28 weeks; very preterm is defined as 28 to 31 completed weeks; moderate-late preterm is defined as 32 to 36 completed weeks; term is defined as 37 to 41 completed weeks; and post-term is defined as ≥42 weeks.

Table S3. Numbers of individuals with psychiatric diagnosis and psychotropic medication use stratified by gestational age or size for gestational age (All live singleton spontaneous delivery births between 1996 and 2014 in Finland followed until 2018 (N=819 764)

**Variables (ICD-10 codes) Psychiatric disorders**	**< 32 weeks & SGA**	**32 to 36 weeks & SGA**	**37 to 41 weeks & AGA**	**< 32 weeks & LGA**	**32 to 36 weeks & LGA**
N	738	2496	724345	363	1696
Any F-diagnosis (F00-F99)	300 (40,65)	602 (24.12)	118004 (16.29)	127 (34.99)	393 (23.17)
Psychotic disorders (F20-F29)	1 (0.14)	6 (0.24)	1931 (0.27)	1 (0.28)	6 (0.35)
Mood disorders (F30-39, F92)	25 (3.39)	115 (4.61)	27991 (3.86)	20 (5.51)	81 (4.78)
Anxiety disorders (F40-43, F93)	61 (8.27)	180 (7.21)	36537 (5.04)	35 (9.64)	121 (7.13)
Eating disorders (F50)	10 (1.36)	17 (0.68)	4310 (0.60)	5 (1.38)	10 (0.59)
Sleeping disorders (F51)	4 (0.54)	22 (0.88)	4356 (0.60)	8 (2.20)	18 (1.06)
Personality disorders (F60-69)	3 (0.41)	11 (0.44)	2187 (0.30)	3 (0.83)	11 (0.65)
Intellectual disabilities (F70-79)	41 (5.56)	91 (3.65)	4877 (0.67)	14 (3.86)	26 (1.53)
SDD (F80-83)	220 (29.81)	329 (13.18)	36749 (5.07)	71 (19.56)	161 (9.49)
ASD (F84)	20 (2.71)	50 (2.00)	7356 (1.02)	9 (2.48)	27 (1.59)
ADHD (F90)	57 (7.72)	123 (4.93)	18492 (2.55)	20 (5.51)	68 (4.07)
Conduct disorders (F91)	0 (0.00)	14 (0.56)	3189 (0.44)	1 (0.28)	12 (0.72)
Other emotional & behavioural disorders (F98)	92 (12.47)	126 (5.05)	18627 (2.57)	25 (6.89)	90 (5.31)
**Psychotropic medication**					
Any medication (N05-N06)	68 (9.21)	224 (8.97)	35508 (4.90)	42 (11.57)	114 (6.72)
Anxiolytics, hypnotics and sedatives (N05)	43 (5.83)	152 (6.09)	23644 (3.26)	27 (7.44)	72 (4.25)
Antidepressants (N06A)	9 (1.22)	30 (1.20)	8303 (1.15)	9 (2.48)	24 (1.42)
Stimulants (N06B)	26 (3.52)	78 (3.13)	10349 (1.43)	14 (3.86)	40 (2.36)

Data are presented as No. (%). Abbreviations: SGA, small for gestational age; AGA, appropriate for gestational age; LGA, large for gestational age; SDD, specific developmental disorders; ASD, autism spectrum disorder; and ADHD, attention-deficit/hyperactivity disorder.

Table S4. Numbers of individuals with psychiatric diagnosis stratified by sex, gestational age, and size for gestational age (All live singleton spontaneous delivery births between 1996 and 2014 in Finland followed until 2018 (N=819 764)

**Exposures**	**Any F-diagnosis**	**Psychotic disorders**	**Mood disorders**	**Anxiety disorders**	**Eating disorders**	**Sleeping disorders**	**Personality disorders**	**Intellectual disabilities**	**SDD**	**ASD**	**ADHD**	**Conduct disorders**	**Other disorders**	**Feeding/ eating disorders (F98.2-F98.3)**
**Boys (n=418 335)**	76773	1151	13778	17661	712	2750	750	4000	31354	6652	17230	2434	13134	2145
**Gestational age, weeks**														
Term (Reference)	69375 (17.98)	1026 (0.27)	12575 (3.26)	16021 (4.15)	639 (0.17)	2480 (0.64)	672 (0.17)	3423 (0.89)	27623 (7.16)	5981 (1.55)	15477 (4.01)	2214 (0.57)	11749 (3.04)	1865 (0.50)
< 28 weeks	333 (45.12)	3 (0.41)	33 (4.47)	54 (7.32)	4 (0.54)	6 (0.81)	7 (0.95)	50 (6.78)	319 (43.22)	41 (5.56)	100 (13.55)	3 (0.41)	67 (9.08)	5 (0.76)
28 to 31 weeks	617 (33.73)	8 (0.44)	60 (3.28)	112 (6.12)	10 (0.55)	19 (1.04)	4 (0.22)	80 (4.37)	438 (23.95)	64 (3.50)	149 (8.15)	13 (0.71)	123 (6.72)	13 (0.86)
32 to 36 weeks	3889 (22.49)	66 (0.38)	635 (.67)	873 (5.05)	40 (0.23)	155 (0.90)	50 (0.29)	316 (1.83)	1934 (11.18)	336 (1.94)	950 (5.49)	125 (0.72)	797 (4.61)	59 (0.45)
≥42 weeks	2203 (19.91)	41 (0.37)	417 (3.77)	509 (4.60)	14 (0.13)	80 (0.72)	16 (0.14)	106 (0.96)	896 (8.10)	197 (1.78)	487 (4.40)	68 (0.61)	353 (3.19)	50 (0.46)
**Size for gestational age**														
AGA (Reference)	71768 (18.13)	1086 (0.27)	12897 (3.26)	16528 (4.17)	661 (0.17)	2575 (0.65)	685 (0.17)	3528 (0.89)	28888 (7.30)	6177 (1.56)	16010 (4.04)	2276 (0.57)	12200 (3.08)	2007 (0.51)
SGA	2783 (23.39)	30 (0.25)	499 (4.19)	639 (5.37)	39 (0.33)	89 (0.75)	41 (0.34)	347 (2.92)	1540 (12.94)	293 (2.46)	781 (6.56)	86 (0.72)	578 (4.86)	79 (0.66)
LGA	1866 (20.71)	28 (0.31)	324 (3.60)	402 (4.46)	7 (0.08)	76 (0.84)	23 (0.26)	100 (1.11)	782 (8.68)	149 (1.65)	372 (4.13)	61 (0.68)	311 (3.45)	51 (0.57)
**Girls (n=401 429)**	60390	1076	18583	24643	4227	2314	1850	2379	13366	2067	4649	1281	8977	2001
**Gestational age, weeks**														

Term (Reference)	55404 (14.83)	1003 (0.27)	17041 (4.56)	22562 (6.04)	3902 (1.04)	2128 (0.57)	1671 (0.45)	2029 (0.54)	11784 (3.15)	1847 (0.49)	4181 (1.12)	1171 (0.31)	8064 (2.16)	1940 (0.50)
< 28 weeks	239 (36.54)	0 (0.00)	42 (6.42)	66 (10.09)	9 (1.38)	3 (0.46)	15 (2.29)	41 (6.27)	206 (31.50)	19 (2.91)	26 (3.98)	8 (1.22)	67 (10.24)	6 (0.81)
28 to 31 weeks	404 (26.84)	3 (0.20)	94 (6.25)	141 (9.37)	21 (1.40)	11 (0.73)	9 (0.60)	43 (2.86)	196 (13.02)	11 (0.73)	41 (2.72)	8 (0.53)	114 (7.57)	10 (0.55)
32 to 36 weeks	2360 (17.82)	38 (0.29)	695 (5.25)	1013 (7.65)	157 (1.19)	106 (0.80)	86 (0.65)	188 (1.42)	736 (5.56)	108 (0.82)	221 (1.67)	56 (0.42)	421 (3.18)	133 (0.77)
≥42 weeks	1721 (15.77)	27 (0.25)	621 (5.69)	752 (6.89)	123 (1.13)	61 (0.56)	56 (0.51)	67 (0.61)	392 (3.59)	69 (0.63)	155 (1.42)	34 (0.31)	271 (2.48)	48 (0.43)
**Size for gestational age**														
AGA (Reference)	56438 (14.87)	1013 (0.27)	17411 (4.59)	23089 (6.08)	3982 (1.05)	2165 (0.57)	1716 (0.45)	2056 (0.54)	12140 (3.20)	1905 (0.5)	4311 (1.14)	1194 (0.31)	8292 (2.18)	1863 (0.49)
SGA	2125 (19.19)	36 (0.33)	624 (5.64)	847 (7.65)	125 (1.13)	87 (0.79)	75 (0.68)	251 (2.27)	828 (7.48)	97 (0.88)	221 (2.00)	52 (0.47)	415 (3.75)	72 (0.65)
LGA	1565 (16.92)	22 (0.24)	458 (4.95)	598 (6.47)	105 (1.14)	57 (0.62)	46 (0.50)	61 (0.66)	346 (3.74)	52 (0.56)	92 (0.99)	31 (0.34)	230 (2.49)	57 (0.62)

Data are presented as No. (%). Abbreviations: Term, individuals born between 37 to 41 completed weeks; AGA, appropriate for gestational age; SGA, small for gestational age; LGA, large for gestational age; SDD, specific developmental disorders; ASD, autism spectrum disorder; and ADHD, attention-deficit/hyperactivity disorder.

Extremely preterm is defined as < 28 weeks; very preterm is defined as 28 to 31 completed weeks; moderate-late preterm is defined as 32 to 36 completed weeks; term is defined as 37 to 41 completed weeks; and post-term is defined as ≥42 weeks.

Table S5. Crude hazard ratios for neurodevelopmental and psychiatric disorders in individuals as a function of gestational age and size for gestational age (All live singleton spontaneous delivery births between 1996 and 2014 in Finland followed until 2018, N=819 764)

**Exposures**	**Any F-diagnosis**	**Psychotic disorders**	**Mood disorders**	**Anxiety disorders**	**Eating disorders**	**Sleeping disorders**	**Personality disorders**	**Intellectual disabilities**	**SDD**	**ASD**	**ADHD**	**Conduct disorders**	**Other disorders**
N	137163	2227	32361	42304	4939	5064	2600	6379	44720	8719	21879	3715	22111
**Gestational age, weeks**													
**Term (Reference)**	1.00	1.00	1.00	1.00	1.00	1.00	1.00	1.00	1.00	1.00	1.00	1.00	1.00
< 28 weeks	3.43 (3.16-3.72)	1.26 (0.41-3.89)	2.13 (1.70-2.67)	2.57 (2.15-3.07)	2.40 (1.39-4.12)	1.33 (0.69-2.56)	7.92 (5.21-12.06)	11.81 (9.60-14.53)	9.82 (9.01-10.71)	5.70 (4.42-7.34)	4.91 (4.12-5.85)	2.52 (1.39-4.56)	5.01 (4.22-5.93)
28 to 31 weeks	2.12 (1.99-2.25)	1.46 (0.81-2.63)	1.39 (1.19-1.63)	1.75 (1.55-1.98)	1.82 (1.28-2.60)	1.63 (1.14-2.34)	1.49 (0.86-2.56)	5.73 (4.79-6.85)	4.16 (3.84-4.50)	2.49 (1.98-3.12)	2.44 (2.12-2.82)	1.56 (1.01-2.39)	3.09 (2.72-3.51)
32 to 36 weeks	1.28 (1.24-1.31)	1.30 (1.07-1.58)	1.14 (1.08-1.21)	1.24 (1.19-1.30)	1.10 (0.95-1.27)	1.43 (1.27-1.62)	1.47 (1.24-1.75)	2.34 (2.14-2.57)	1.73 (1.66-1.80)	1.44 (1.31-1.59)	3.06 (2.74-3.42)	1.79 (1.27-2.54)	1.57 (1.48-1.66)
≥42 weeks	1.02 (0.99-1.06)	1.05 (0.82-1.33)	1.10 (1.03-1.17)	1.03 (0.97-1.09)	0.94 (0.80-1.12)	1.01 (0.86-1.20)	0.97 (0.76-1.22)	1.05 (0.90-1.22)	1.08 (1.02-1.14)	1.11 (0.98-1.25)	1.47 (1.39-1.56)	1.31 (1.13-1.52)	1.03 (0.95-1.11)
**Size for gestational age**													
**AGA (Reference)**	1.00	1.00	1.00	1.00	1.00	1.00	1.00	1.00	1.00	1.00	1.00	1.00	1.00
SGA	1.35 (1.32-1.39)	1.15 (0.9-1.47)	1.35 (1.27-1.44)	1.36 (1.29-1.44)	1.29 (1.11-1.51)	1.31 (1.13-1.52)	1.77 (1.47-2.13)	3.75 (3.45-4.09)	2.03 (1.95-2.12)	1.70 (1.54-1.88)	1.52 (1.42-1.62)	1.23 (1.04-1.46)	1.71 (1.60-1.82)
LGA	1.08 (1.04-1.12)	0.88 (0.66-1.16)	0.97 (0.90-1.04)	0.96 (0.90-1.02)	0.90 (0.74-1.08)	1.13 (0.95-1.34)	1.06 (0.83-1.34)	1.17 (1.00-1.37)	1.12 (1.06-1.19)	1.01 (0.87-1.16)	1.04 (0.95-1.14)	1.15 (0.93-1.41)	1.07 (0.98-1.17)
**Birth outcomes combined**													
**Term & AGA (Reference)**	1.00	1.00	1.00	1.00	1.00	1.00	1.00	1.00	1.00	1.00	1.00	1.00	1.00
< 32 weeks & SGA	3.21 (2.86-3.59)	0.73 (0.10-5.19)	1.25 (0.84-1.84)	2.30 (1.79-2.96)	3.22 (1.73-5.99)	1.08 (0.41-2.88)	1.90 (0.61-5.90)	9.29 (6.83-12.63)	7.24 (6.34-8.26)	3.39 (2.19-5.25)	3.90 (3.01-5.06)	NA	6.06 (4.94-7.44)
32 to 36 weeks & SGA	1.55 (1.43-1.68)	0.98 (0.44-2.18)	1.29 (1.07-1.55)	1.54 (1.33-1.78)	1.25 (0.78-2.02)	1.52 (1.00-2.30)	1.54 (0.85-2.79)	5.08 (4.13-6.25)	2.62 (2.35-2.92)	2.03 (1.54-2.68)	1.84 (1.54-2.20)	1.23 (0.73-2.07)	2.02 (1.70-2.41)

< 32 weeks & LGA	2.35 (1.97-2.79)	1.06 (0.15-7.51)	1.49 (0.96-2.3)	2.01 (1.44-2.80)	2.40 (1.00-5.78)	3.81 (1.90-7.62)	2.70 (0.87-8.38)	5.56 (3.29-9.39)	4.09 (3.24-5.16)	2.62 (1.36-5.04)	2.41 (1.55-3.74)	NA	2.88 (1.95-4.26)
32 to 36 weeks & LGA	1.42 (1.28-1.56)	1.28 (0.57-2.85)	1.20 (0.97-1.49)	1.37 (1.15-1.64)	0.97 (0.52-1.80)	1.75 (1.10-2.79)	2.02 (1.12-3.65)	1.98 (1.34-2.94)	1.78 (1.52-2.08)	1.49 (1.02-2.19)	1.60 (1.26-2.03)	1.40 (0.81-2.83)	2.05 (1.67-2.52)

Data are presented as adjusted hazard ratios (HRs) and (95% CI). Abbreviations: Term, individuals born between 37 to 41 completed weeks; AGA, appropriate for gestational age; SGA, small for gestational age; LGA, large for gestational age; SDD, specific developmental disorders; ASD, autism spectrum disorder; and ADHD, attention-deficit/hyperactivity disorder.

Extremely preterm is defined as < 28 weeks; very preterm is defined as 28 to 31 completed weeks; moderate-late preterm is defined as 32 to 36 completed weeks; term is defined as 37 to 41 completed weeks; and post-term is defined as ≥42 weeks.

Table S6. Sibling analysis: Risk for neurodevelopmental and psychiatric disorders in second-born child after exposure to premature birth (before 37 gestational weeks), as estimated by matched sibling pair analysis. All singleton sibling pairs (n=299 331) among the 819 764 births (born 1996-2014) were included and followed-up until 2018

Exposure: Preterm birth	Any F-diagnosis	Mood disorders	Anxiety disorders	Intellectual disabilities	SDD	ADHD	Other disorders
**Model 1**							
None of the sibling pairs were exposed (Reference)	1.00	1.00	1.00	1.00	1.00	1.00	1.00
Exposure in the first, but not during the second pregnancy	1.09 (1.03-1.15)	1.05 (0.96-1.14)	1.14 (1.06-1.23)	1.37 (1.17-1.60)	1.11 (1.04-1.18)	1.15 (1.04-1.26)	1.00 (0.91-1.10)
Exposure in the second, but not during first pregnancy	1.51 (1.44-1.59)	1.09 (1.00-1.20)	1.17 (1.08-1.26)	2.38 (2.08-2.72)	1.73 (1.63-1.84)	1.61 (1.47-1.76)	1.43 (1.30-1.57)
Both siblings in the pair were exposed	1.57 (1.45-1.69)	1.17 (1.02-1.35)	1.35 (1.21-1.52)	2.27 (1.85-2.79)	1.72 (1.57-1.87)	1.44 (1.25-1.67)	1.57 (1.38-1.80)
**Model 2**							
None of the sibling pairs were exposed (Reference)	1.00	1.00	1.00	1.00	1.00	1.00	1.00
Exposure in the first, but not during the second pregnancy	1.07 (1.01-1.13)	1.03 (0.94-1.12)	1.12 (1.05-1.21)	1.34 (1.15-1.57)	1.08 (1.01-1.15)	1.11 (1.01-1.22)	0.99 (0.90-1.09)
Exposure in the second, but not during first pregnancy	1.50 (1.42-1.58)	1.09 (0.99-1.19)	1.16 (1.07-1.25)	2.36 (2.06-2.70)	1.72 (1.62-1.82)	1.59 (1.45-1.74)	1.42 (1.29-1.56)
Both siblings in the pair were exposed	1.52 (1.41-1.65)	1.14 (0.99-1.31)	1.31 (1.17-1.47)	2.20 (1.79-2.70)	1.66 (1.52-1.81)	1.38 (1.19-1.59)	1.54 (1.35-1.76)

Data are presented as adjusted hazard ratios (HRs) and (95% CI). Abbreviations: SDD, specific developmental disorders; ASD, autism spectrum disorder; and ADHD, attention-deficit/hyperactivity disorder.
Model 1: Adjusted for offspring sex, birth year, maternal age at delivery, mother's country of birth (Finland or not), mother married at birth (yes/no), mother SES, maternal smoking (yes/no), parity (0 or ≥1), maternal obesity (yes/no), maternal inpatient and outpatient psychiatric history (yes/no), maternal N05/N06 purchase during pregnancy, maternal systemic inflammatory disease (yes/no), and intra-pregnancy interval.
Model 2: Adjusted for the variables in Model 1 and presence of the studied F diagnosis or psychotropic medication in the first child.

Table S7. Sibling analysis: Risk for neurodevelopmental and psychiatric disorders in second-born child after the exposure being born small for gestational age (SGA), as estimated by sibling pair analysis. All singleton sibling pairs (n=299 331) among the 819 764 births (born 1996-2014) were included and followed-up until 2018

Exposure: Small for gestational age (SGA)	Any F-diagnosis	Mood disorders	Anxiety disorders	Intellectual disabilities	SDD	ADHD	Other disorders
**Model 1**							
None of the sibling pairs were exposed (Reference)	1.00	1.00	1.00	1.00	1.00	1.00	1.00
Exposure in the first, but not in the second pregnancy	1.10 (1.01-1.19)	0.95 (0.83-1.09)	1.02 (0.91-1.14)	1.19 (0.70-2.03)	1.17 (1.07-1.29)	1.02 (0.89-1.18)	1.21 (1.06-1.38)
Exposure in the second, but not in the first pregnancy	1.65 (1.50-1.80)	1.30 (1.10-1.53)	1.27 (1.10-1.46)	2.15 (1.23-3.73)	1.97 (1.79-2.16)	1.22 (1.02-1.45)	1.62 (1.38-1.89)
Both siblings in the pair were exposed	1.89 (1.60-2.24)	1.50 (1.10-2.03)	1.56 (1.21-2.01)	4.93 (2.33-10.42)	2.08 (1.73-2.51)	2.03 (1.55-2.67)	1.54 (1.12-2.13)
**Model 2**							
None of the sibling pairs were exposed (Reference)	1.00	1.00	1.00	1.00	1.00	1.00	1.00
Exposure in the first, but not in the second pregnancy	1.07 (0.98-1.16)	0.93 (0.81-1.06)	0.99 (0.88-1.11)	1.16 (0.68-1.98)	1.14 (1.04-1.25)	0.98 (0.85-1.14)	1.18 (1.03-1.35)
Exposure in the second, but not in the first pregnancy	1.62 (1.48-1.77)	1.28 (1.09-1.51)	1.25 (1.08-1.43)	2.12 (1.22-3.69)	1.93 (1.75-2.12)	1.18 (0.99-1.41)	1.59 (1.36-1.87)
Both siblings in the pair were exposed	1.77 (1.50-2.10)	1.39 (1.02-1.88)	1.45 (1.13-1.87)	4.59 (2.17-9.72)	1.96 (1.63-2.35)	1.85 (1.41-2.43)	1.47 (1.06-2.03)

Data are presented as adjusted hazard ratios (HRs) and (95% CI). Abbreviations: SDD, specific developmental disorders; ASD, autism spectrum disorder; and ADHD, attention-deficit/hyperactivity disorder.
Model 1: Adjusted for offspring sex, birth year, maternal age at delivery, mother's country of birth (Finland or not), mother married at birth (yes/no), mother SES, maternal smoking (yes/no), parity (0 or ≥1), maternal obesity (yes/no), maternal inpatient and outpatient psychiatric history (yes/no), maternal N05/N06 purchase during pregnancy, maternal systemic inflammatory disease (yes/no), and intra-pregnancy interval.
Model 2: Adjusted for the variables in Model 1 and presence of the studied F diagnosis or psychotropic medication in the first child.

Table S8. Boys: Adjusted hazard ratios (HRs) for neurodevelopmental and psychiatric disorders in boys in relation to gestational age and size for gestational age (All live singleton spontaneous delivery births between 1996 and 2014 in Finland followed until 2018 (N=418 335)

**Exposures**	**Any F-diagnosis**	**Psychotic disorders**	**Mood disorders**	**Anxiety disorders**	**Eating disorders**	**Sleeping disorders**	**Personality disorders**	**Intellectual disabilities**	**SDD**	**ASD**	**ADHD**	**Conduct disorders**	**Other disorders**	**Feeding/eating disorders**
**Gestational age, weeks**														
**Term (Reference)**	1.00	1.00	1.00	1.00	1.00	1.00	1.00	1.00	1.00	1.00	1.00	1.00	1.00	1.00
< 28 weeks	3.32 (2.98-3.69)	2.44 (0.79-7.56)	1.78 (1.27-2.51)	2.27 (1.74-2.97)	4.05 (1.52-10.83)	1.38 (0.62-3.07)	6.84 (3.25-14.43)	8.97 (6.78-11.86)	7.54 (6.75-8.42)	4.28 (3.15-5.82)	4.52 (3.71-5.50)	0.92 (0.30-2.87)	3.86 (3.04-4.91)	1.73 (0.77-3.87)
28 to 31 weeks	2.08 (1.92-2.25)	2.05 (1.02-4.10)	1.14 (0.89-1.47)	1.64 (1.36-1.97)	3.58 (1.91-6.68)	1.66 (1.06-2.61)	1.38 (0.52-3.68)	5.38 (4.31-6.72)	3.75 (3.42-4.13)	2.28 (1.78-2.91)	2.29 (1.95-2.69	1.39 (0.80-2.39)	2.45 (2.05-2.92)	1.14 (0.61-2.13)
< 32 weeks	2.43 (2.28-2.59)	2.14 (1.18-3.87)	1.31 (1.07-1.61)	1.80 (1.54-2.10)	3.70 (2.18-6.29)	1.58 (1.07-2.35)	2.80 (1.54-5.09)	6.36 (5.33-7.58)	4.76 (4.43-5.12)	2.79 (2.30-3.39)	2.85 (2.52-3.23)	1.27 (0.77-2.07)	2.81 (2.44-3.24)	1.31 (0.80-2.14)
32 to 36 weeks	1.25 (1.21-1.29)	1.47 (1.15-1.89)	1.11 (1.02-1.20)	1.19 (1.11-1.27)	1.36 (0.99-1.88)	1.35 (1.15-1.59)	1.62 (1.21-2.16)	2.06 (1.84-2.32)	1.58 (1.51-1.65)	1.17 (1.05-1.31)	1.35 (1.27-1.44)	1.23 (1.03-1.48)	1.51 (1.4-1.62)	1.55 (1.30-1.85)
≥ 42 weeks	1.02 (0.97-1.06)	1.27 (0.93-1.74)	1.04 (0.94-1.15)	1.00 (0.92-1.10)	0.69 (0.41-1.17)	1.06 (0.85-1.33)	0.73 (0.45-1.2)	1.04 (0.86-1.27)	1.09 (1.02-1.17)	1.01 (0.88-1.17)	1.02 (0.93-1.11)	0.97 (0.76-1.23)	0.98 (0.88-1.09)	0.87 (0.65-1.16)
**Size for gestational age**														
**AGA (Reference)**	1.00	1.00	1.00	1.00	1.00	1.00	1.00	1.00	1.00	1.00	1.00	1.00	1.00	1.00
SGA	1.29 (1.24-1.34)	1.01 (0.7-1.45)	1.19 (1.09-1.31)	1.20 (1.11-1.30)	2.00 (1.44-2.76)	1.08 (0.87-1.33)	1.84 (1.34-2.53)	3.29 (2.94-3.67)	1.73 (1.64-1.82)	1.42 (1.26-1.59)	1.46 (1.36-1.57)	1.14 (0.92-1.42)	1.53 (1.41-1.67)	1.32 (1.05-1.66)
LGA	1.06 (1.01-1.11)	0.94 (0.65-1.37)	1.07 (0.95-1.19)	1.03 (0.93-1.14)	0.41 (0.19-0.85)	1.29 (1.03-1.63)	1.37 (0.90-2.07)	1.15 (0.95-1.41)	1.18 (1.10-1.27)	1.10 (0.93-1.29)	1.10 (0.99-1.22)	1.19 (0.92-1.54)	1.14 (1.02-1.27)	1.13 (0.85-1.49)
**Birth outcomes combined**														
**Term & AGA (Reference)**	1.00	1.00	1.00	1.00	1.00	1.00	1.00	1.00	1.00	1.00	1.00	1.00	1.00	1.00

< 32 weeks & SGA	3.56 (3.05-4.15)	1.85 (0.26-13.14)	0.89 (0.40-1.97)	2.12 (1.37-3.29)	9.29 (3.48-24.82)	1.03 (0.26-4.13)	2.71 (0.38-19.31)	9.86 (6.60-14.72)	7.45 (6.30-8.81)	3.43 (2.10-5.61)	4.20 (3.06-5.75)	NA	4.92 (3.59-6.74)	0.00 (0.00-3.94)
32 to 36 weeks & SGA	1.48 (1.34-1.64)	0.61 (0.15-2.43)	1.14 (0.87-1.50)	1.23 (0.97-1.55)	2.18 (0.90-5.25)	1.51 (0.89-2.55)	0.82 (0.21-3.29)	4.18 (3.18-5.5)	2.17 (1.89-2.48)	1.42 (1.01-1.99)	1.63 (1.33-2.00)	0.86 (0.41-1.81)	2.06 (1.67-2.55)	2.86 (1.82-4.49)
< 32 weeks & LGA	2.09 (1.68-2.61)	0.00 (0.00-12.00)	0.95 (0.45-1.98)	2.28 (1.48-3.49)	2.52 (0.35-17.92)	2.73 (1.03-7.28)	2.05 (0.29-14.62)	4.16 (2.08-8.33)	3.36 (2.52-4.49)	2.08 (0.99-4.37)	2.23 (1.37-3.64)	NA	2.17 (1.26-3.73)	3.04 (0.98-9.42)
32 to 36 weeks & LGA	1.26 (1.10-1.44)	1.20 (0.39-3.72)	1.24 (0.91-1.71)	1.37 (1.05-1.79)	1.23 (0.31-4.92)	1.62 (0.87-3.01)	4.17 (1.98-8.77)	1.56 (0.93-2.64)	1.42 (1.16-1.73)	1.13 (0.7-1.82)	1.41 (1.07-1.85)	1.70 (0.88-3.27)	1.89 (1.44-2.47)	1.98 (1.03-3.82)

Data are presented as adjusted hazard ratios (HRs) and 95% CI. Abbreviations: Term, individuals born between 37 to 41 completed weeks; AGA, appropriate for gestational age; SGA, small for gestational age; LGA, large for gestational age; SDD, specific developmental disorders; ASD, autism spectrum disorder; and ADHD, attention-deficit/hyperactivity disorder.

Extremely preterm is defined as < 28 weeks; very preterm is defined as 28 to 31 completed weeks; moderate-late preterm is defined as 32 to 36 completed weeks; term is defined as 37 to 41 completed weeks; and post-term is defined as ≥42 weeks.
The analyses were adjusted for the birth year of a child maternal age at child birth, parity (0 or ≥1), maternal cohabitation status at child birth (yes/no), maternal country of origin (Finland or other), maternal occupation, smoking during pregnancy (yes/no), maternal obesity (*ICD-10* codes; E65-66, yes/no), maternal in-patient (from 1987) and out-patient (from 1998) psychiatric history (yes/no), maternal use of anti-psychotropic medication during pregnancy (ATC codes; N05/N06, yes/no) and maternal systemic inflammatory disease (*ICD-10* codes M30-M36; yes/no).

Table S9. Girls: Adjusted hazard ratios (HRs) for neurodevelopmental and psychiatric disorders in girls in relation to gestational age and size for gestational age (All live singleton spontaneous delivery births between 1996 and 2014 in Finland followed until 2018 (N=401 429)

Exposures	Any F-diagnosis	Psychotic disorders	Mood disorders	Anxiety disorders	Eating disorders	Sleeping disorders	Personality disorders	Intellectual disabilities	SDD	ASD	ADHD	Conduct disorders	Other disorders	Feeding/ eating disorders
**Gestational age, weeks**														
**Term (Reference)**	1.00	1.00	1.00	1.00	1.00	1.00	1.00	1.00	1.00	1.00	1.00	1.00	1.00	1.00
< 28 weeks	3.34 (2.94-3.79)	NA	2.15 (1.59-2.91)	2.44 (1.92-3.11)	1.99 (1.04-3.83)	0.97 (0.31-3.00)	7.87 (4.73-13.10)	13.98 (10.25-19.06)	12.52 (10.90-14.37)	7.13 (4.53-11.21)	4.90 (3.33-7.21)	5.60 (2.79-11.23)	6.03 (4.74-7.68)	1.68 (0.69-4.04)
28 to 31 weeks	1.90 (1.72-2.10)	0.81 (0.26-2.51)	1.40 (1.14-1.71)	1.58 (1.34-1.86)	1.41 (0.92-2.17)	1.26 (0.70-2.28)	1.30 (0.67-2.50)	5.33 (3.94-7.22)	4.33 (3.76-4.99)	1.45 (0.80-2.63)	2.48 (1.82-3.37)	1.71 (0.85-3.43)	3.66 (3.05-4.41)	1.82 (1.05-3.14)
< 32 weeks	2.30 (2.12-2.48)	0.63 (0.20-1.95)	1.57 (1.32-1.85)	1.78 (1-55-2.04)	1.55 (1.08-2.22)	1.18 (0.70-2.00)	2.72 (1.81-4.07)	7.64 (6.13-9.51)	6.51 (5.89-7.20)	2.94 (2.05-4.21)	3.07 (2.41-3.91)	2.62 (1.60-4.30)	4.29 (3.70-4.97)	1.77 (1.12-2.83)
32 to 36 weeks	1.18 (1.13-1.23)	1.06 (0.77-1.47)	1.10 (1.02-1.19)	1.21 (1.13-1.28)	1.10 (0.94-1.29)	1.32 (1.09-1.61)	1.35 (1.09-1.68)	2.57 (2.21-2.99)	1.75 (1.62-1.88)	1.53 (1.26-1.86)	1.41 (1.23-1.61)	1.26 (0.97-1.65)	1.43 (1.30-1.58)	0.90 (0.69-1.17)
≥42 weeks	0.96 (0.91-1.01)	0.81 (0.56-1.19)	1.10 (1.01-1.19)	1.01 (0.94-1.08)	0.95 (0.79-1.14)	0.90 (0.7-1.16)	0.99 (0.75-1.29)	1.08 (0.85-1.38)	1.09 (0.99-1.2)	1.14 (0.90-1.45)	1.15 (0.98-1.35)	0.87 (0.62-1.23)	1.07 (0.94-1.20)	0.92 (0.69-1.22)
**Size for gestational age**														
**AGA (Reference)**	1.00	1.00	1.00	1.00	1.00	1.00	1.00	1.00	1.00	1.00	1.00	1.00	1.00	1.00
SGA	1.30 (1.25-1.36)	1.34	1.18 (1.09-1.28)	1.20	1.13	1.29	1.38	4.20	2.27 (2.12-2.44)	1.62	1.57	1.34	1.66	1.36 (1.08-1.73)

		(0.96-1.87)		(1.12-1.29)	(0.94-1.35)	(1.04-1.60)	(1.09-1.74)	(3.68-4.80)		(1.32-1.98)	(1.38-1.80)	(1.02-1.78)	(1.5-1.83)	
LGA	1.04 (0.99-1.09)	0.74 (0.48-1.13)	0.98 (0.89-1.08)	0.99 (0.91-1.07)	0.94 (0.77-1.14)	1.07 (0.82-1.39)	0.99 (0.74-1.32)	1.11 (0.86-1.43)	1.15 (1.03-1.28)	1.12 (0.85-1.47)	0.91 (0.74-1.12)	1.08 (0.76-1.55)	1.14 (1.00-1.30)	1.24 (0.95-1.62)
**Birth outcomes combined**														
**Term & AGA (Reference)**	1.00	1.00	1.00	1.00	1.00	1.00	1.00	1.00	1.00	1.00	1.00	1.00	1.00	1.00
< 32 weeks & SGA	2.79 (2.36-3.30)	0.00 (0-16.89)	1.30 (0.83-2.03)	2.03 (1.50-2.76)	1.74 (0.78-3.88)	0.92 (0.23-3.68)	1.26 (0.32-5.06)	8.48 (5.26-13.68)	7.70 (6.20-9.55)	2.08 (0.78-5.55)	4.60 (2.90-7.32)	NA	6.97 (5.32-9.13)	1.10 (0.27-4.41)
32 to 36 weeks & SGA	1.46 (1.29-1.66)	1.42 (0.53-3.79)	1.17 (0.92-1.50)	1.48 (1.23-1.79)	1.02 (0.58-1.80)	1.14 (0.57-2.29)	1.55 (0.80-2.98)	5.77 (4.20-7.93)	3.11 (2.59-3.74)	2.54 (1.55-4.15)	2.16 (1.51-3.09)	1.82 (0.87-3.83)	1.57 (1.15-2.14)	1.07 (0.48-2.39)
< 32 weeks & LGA	1.99 (1.49-2.64)	2.59 (0.36-18.41)	1.78 (1.03-3.06)	1.46 (0.86-2.46)	2.53 (0.95-6.74)	4.37 (1.64-11.65)	2.82 (0.71-11.28)	6.57 (2.95-14.64)	5.02 (3.39-7.43)	2.46 (0.61-9.83)	2.27 (0.85-6.05)	1.90 (.27-13.53)	3.58 (2.04-6.32)	1.38 (0.19-9.83)
32 to 36 weeks & LGA	1.41 (1.22-1.63)	1.26 (0.41-3.91)	1.08 (0.80-1.46)	1.27 (1.00-1.61)	0.87 (0.44-1.75)	1.67 (0.83-3.34)	0.97 (0.36-2.58)	2.29 (1.27-4.15)	2.35 (1.82-3.02)	2.15 (1.12-4.13)	1.78 (1.09-2.90)	1.15 (0.37-3.56)	2.05 (1.48-2.85)	1.33 (0.55-3.20)

Data are presented as adjusted hazard ratios (HRs) and 95% CI. Abbreviations: Term, individuals born between 37 to 41 completed weeks; AGA, appropriate for gestational age; SGA, small for gestational age; LGA, large for gestational age; SDD, specific developmental disorders; ASD, autism spectrum disorder; and ADHD, attention-deficit/hyperactivity disorder.

Extremely preterm is defined as < 28 weeks; very preterm is defined as 28 to 31 completed weeks; moderate-late preterm is defined as 32 to 36 completed weeks; term is defined as 37 to 41 completed weeks; and post-term is defined as ≥42 weeks.
The analyses were adjusted for the birth year of a child maternal age at child birth, parity (0 or ≥1), maternal cohabitation status at child birth (yes/no), maternal country of origin (Finland or other), maternal occupation, smoking during pregnancy (yes/no), maternal obesity (*ICD-10* codes; E65-66, yes/no), maternal in-patient (from 1987) and out-patient (from 1998) psychiatric history (yes/no), maternal use of psychotropic medication during pregnancy (ATC codes; N05/N06, yes/no) and maternal systemic inflammatory disease (*ICD-10* codes M30-M36; yes/no).

Table S10. Numbers of individuals with psychotropic medication use stratified by sex, gestational age, and size for gestational age (All live singleton spontaneous delivery births between 1996 and 2014 in Finland followed until 2018, N=819 764)

**Exposures**	**Any psychotropic medication**	**Antipsychotics, anxiolytics, hypnotics and sedatives**	**Antidepressants**	**Stimulants**
**Boys**				
n	24336	15256	3441	10105
**Gestational age, weeks**				
**Term (Reference)**	22050 (5.71)	13823 (3.58)	3157 (0.82)	9114 (2.36)
< 28 weeks	88 (11.92)	50 (6.78)	9 (1.22)	45 (6.10)
28 to 31 weeks	202 (11.04)	131 (7.16)	23 (1.26)	94 (5.14)
< 32 weeks	290 (11.9)	181 (11.9)	32 (0.92)	139 (5.50)
32 to 36 weeks	1328 (7.68)	836 (4.83)	139 (0.80)	584 (3.38)
≥ 42 weeks	668 (6.04)	416 (3.76)	113 (1.02)	268 (2.42)
**Size for gestational age**				
**AGA (Reference)**	22738 (5.74)	14263 (3.6)	3218 (0.81)	9415 (2.38)
SGA	988 (8.3)	596 (5.01)	125 (1.05)	460 (3.87)
LGA	610 (6.77)	397 (4.41)	98 (1.09)	230 (2.55)
**Girls**				
n	16953	12188	6115	2115
**Gestational age, weeks**				
**Term (Reference)**	15591 (4.17)	11221 (3)	5646 (1.51)	1906 (0.51)
< 28 weeks	61 (9.33)	44 (6.73)	18 (2.75)	12 (1.83)
28 to 31 weeks	114 (7.57)	88 (5.85)	32 (2.13)	18 (1.20)
< 32 weeks	175 (8.45	132 (6.29)	50 (2.44)	40 (1.51)
32 to 36 weeks	699 (5.28)	496 (3.75)	229 (1.73)	112 (0.85)
≥ 42 weeks	488 (4.47)	339 (3.11)	190 (1.74)	67 (0.61)
**Size for gestational age**				
AGA (Reference)	15927 (4.20)	11458 (3.02)	5756 (1.52)	1962 (0.52)
SGA	599 (5.41)	430 (3.88)	186 (1.68)	103 (0.93)
LGA	427 (4.62)	300 (3.24)	173 (1.87)	50 (0.54)

Data are presented as No. (%). Abbreviations: Term, individuals born between 37 to 41 completed weeks; AGA, appropriate for gestational age; SGA, small for gestational age; and LGA, large for gestational age.

Extremely preterm is defined as < 28 weeks; very preterm is defined as 28 to 31 completed weeks; moderate-late preterm is defined as 32 to 36 completed weeks; and post-term is defined as ≥42 weeks.

Table S11. Hazard ratios for boys' psychotropic medication use in relation to gestational age and size for gestational age (All live singleton spontaneous delivery births between 1996 and 2014 in Finland followed until 2018)

**Exposures**	**Any psychotropic medication**	**Antipsychotics, anxiolytics, hypnotics and sedatives**	**Antidepressants**	**Stimulants**
**Gestational age, weeks**				
**Term (Reference)**	1.00	1.00	1.00	1.00
< 28 weeks	2.89 (2.35-3.57)	2.64 (2.00-3.49)	1.84 (0.96-3.55)	3.43 (2.56-4.59)
28 to 31 weeks	2.24 (1.95-2.57)	2.35 (1.98-2.79)	1.75 (1.16-2.63)	2.44 (1.99-2.99)
< 32 weeks	2.41 (2.14-2.70)	2.42 (2.09-2.80)	1.77 (1.25-2.51)	2.69 (227-3.18)
32 to 36 weeks	1.34 (1.27-1.42)	1.35 (1.26-1.45)	0.96 (0.81-1.13)	1.40 (1.29-1.52)
≥42 weeks	0.96 (0.89-1.03)	0.96 (0.87-1.05)	1.09 (0.9-1.32)	0.91 (0.81-1.03)
**Size for gestational age**				
**AGA (Reference)**	1.00	1.00	1.00	1.00
SGA	1.41 (1.32-1.50)	1.38 (1.27-1.50)	1.25 (1.05-1.50)	1.48 (1.35-1.63)
LGA	1.10 (1.01-1.19)	1.12 (1.02-1.24)	1.14 (0.94-1.40)	1.06 (0.93-1.21)
**Birth outcomes combined**				
**Term & AGA (Reference)**	1.00	1.00	1.00	1.00
< 32 weeks & SGA	2.93 (2.11-4.06)	2.35 (1.48-3.73)	2.35 (0.88-6.28)	3.41 (2.15-5.41)
32 to 36 weeks & SGA	1.93 (1.64-2.27)	2.05 (1.68-2.51)	0.99 (0.55-1.79)	1.78 (1.38-2.30)
< 32 weeks & LGA	2.06 (1.41-3.01)	2.17 (1.37-3.44)	1.21 (0.39-3.76)	2.33 (1.32-4.11)
32 to 36 weeks & LGA	1.32 (1.05-1.67)	1.34 (1.00-1.79)	1.13 (0.59-2.17)	1.52 (1.09-2.14)

Data are presented as adjusted hazard ratios (HRs) and 95% CI. Abbreviations: Term, individuals born between 37 to 41 completed weeks; AGA, appropriate for gestational age; SGA, small for gestational age; and LGA, large for gestational age.

Extremely preterm is defined as < 28 weeks; very preterm is defined as 28 to 31 completed weeks; moderate-late preterm is defined as 32 to 36 completed weeks; and post-term is defined as ≥42 weeks.
The analyses were adjusted for the birth year of a child maternal age at child birth, parity (0 or ≥1), maternal cohabitation status at child birth (yes/no), maternal country of origin (Finland or other), maternal occupation, smoking during pregnancy (yes/no), maternal obesity (*ICD-10* codes; E65-66, yes/no), maternal in-patient (from 1987) and out-patient (from 1998) psychiatric history (yes/no), maternal use of psychotropic medication during pregnancy (ATC codes; N05/N06, yes/no) and maternal systemic inflammatory disease (*ICD-10* codes M30-M36; yes/no).

Psychotropic medications were defined according to the ATC classification system: antipsychotics, anxiolytics, hypnotics and sedatives (ATC groups N05); antidepressants (ATC group N06A); stimulants (ATC group N06B).

Table S12. Hazard ratios for girls' psychotropic medication use in relation to gestational age and size for gestational age (All live singleton spontaneous delivery births between 1996 and 2014 in Finland followed until 2018)

Exposures	Any psychotropic medication	Antipsychotics, anxiolytics, hypnotics and sedatives	Antidepressants	Stimulants
**Gestational age, weeks**				
**Term (Reference)**	1.00	1.00	1.00	1.00
< 28 weeks	3.53 (2.74-4.53)	3.53 (2.62-4.74)	3.06 (1.92-4.85)	5.29 (3.00-9.34)
28 to 31 weeks	1.83 (1.52-2.20)	1.99 (1.61-2.46)	1.35 (0.95-1.91)	2.33 (1.46-3.71)
< 32 weeks	2.20 (1.89-2.55)	2.33 (1.96-2.77)	1.69 (1.28-2.24)	3.01 (2.10-4.31)
32 to 36 weeks	1.22 (1.13-1.32)	1.21 (1.11-1.33)	1.09 (0.95-1.24)	1.56 (1.29-1.89)
≥42 weeks	0.95 (0.87-1.04)	0.92 (0.83-1.03)	1.02 (0.88-1.18)	1.06 (0.83-1.35)
**Size for gestational age**				
**AGA (Reference)**	1.00	1.00	1.00	1.00
SGA	1.28 (1.18-1.39)	1.29 (1.17-1.42)	1.07 (0.92-1.24)	1.62 (1.32-1.97)
LGA	0.98 (0.89-1.08)	0.96 (0.86-1.08)	1.03 (0.89-1.2)	1.05 (0.79-1.39)
**Birth outcomes combined**				
**Term & AGA (Reference)**	1.00	1.00	1.00	1.00
< 32 weeks & SGA	2.37 (1.68-3.36)	2.59 (1.75-3.83)	1.01 (0.42-2.44)	4.74 (2.37-9.5)
32 to 36 weeks & SGA	1.6 (1.28-2)	1.62 (1.24-2.1)	1.02 (0.65-1.59)	3.01 (1.92-4.74)
< 32 weeks & LGA	2.49 (1.5-4.14)	2.15 (1.12-4.13)	2.66 (1.2-5.93)	2.34 (0.59-9.37)
32 to 36 weeks & LGA	1.12 (0.82-1.52)	0.96 (0.65-1.43)	1.02 (0.61-1.69)	1.38 (0.62-3.08)

Data are presented as adjusted hazard ratios (HRs) and 95% CI. Abbreviations: Term, individuals born between 37 to 41 completed weeks; AGA, appropriate for gestational age; SGA, small for gestational age; and LGA, large for gestational age.

Extremely preterm is defined as < 28 weeks; very preterm is defined as 28 to 31 completed weeks; moderate-late preterm is defined as 32 to 36 completed weeks; and post-term is defined as ≥42 weeks.
The analyses were adjusted for the birth year of a child maternal age at child birth, parity (0 or ≥1), maternal cohabitation status at child birth (yes/no), maternal country of origin (Finland or other), maternal occupation (upper white-collar worker, lower white-collar worker, blue-collar worker, other status), smoking during pregnancy (yes/no), maternal obesity (*ICD-10* codes; E65-66, yes/no), maternal in-patient (from 1987) and out-patient (from 1998) psychiatric history (yes/no), maternal use of psychotropic medication during pregnancy (ATC codes; N05/N06, yes/no) and maternal systemic inflammatory disease (*ICD-10* codes M30-M36; yes/no).

Psychotropic medications were defined according to the ATC classification system: antipsychotics, anxiolytics, hypnotics and sedatives (ATC groups N05); antidepressants (ATC group N06A); stimulants (ATC group N06B).

Table S13. Adjusted hazard ratios (HRs) for mood and anxiety disorders in individuals at least 10 years of age in relation to gestational age and size for gestational age (N=561 350, born 1996-2008 followed until 2018).

**Exposures**	**Mood disorders**	**Anxiety disorders**
N	561350	561350
**Gestational age, weeks**		
**Term (Reference)**	1.00	1.00
< 28 weeks	1.24 (1.01-1.51)	1.30 (1.10-1.54)
28 to 31 weeks	1.11 (0.98-1.27)	1.20 (1.08-1.34)
< 32 weeks	1.15 (1.03-1.28)	1.23 (1.12-1.35)
32 to 36 weeks	1.01 (0.96-1.05)	1.05 (1.01-1.09)
≥42 weeks	1.02 (0.97-1.08)	0.96 (0.92-1.01)
**Size for gestational age**		
**AGA (Reference)**	1.00	1.00
SGA	1.09 (1.04-1.15)	1.07 (1.02-1.12)
LGA	1.00 (0.95-1.06)	0.98 (0.93-1.03)
**Birth outcomes combined**		
**Term & AGA (Reference)**	1.00	1.00
< 32 weeks & SGA	0.99 (0.74-1.33)	1.29 (1.03-1.63)
32 to 36 weeks & SGA	0.95 (0.81-1.12)	1.16 (1.01-1.32)
< 32 weeks & LGA	1.22 (0.87-1.71)	1.31 (0.99-1.72)
32 to 36 weeks & LGA	1.03 (0.86-1.24)	1.14 (0.97-1.32)

Data are presented as adjusted hazard ratios (HRs) and 95% CI. Abbreviations: Term, individuals born between 37 to 41 completed weeks; AGA, appropriate for gestational age; SGA, small for gestational age; and LGA, large for gestational age.

Extremely preterm is defined as < 28 weeks; very preterm is defined as 28 to 31 completed weeks; moderate-late preterm is defined as 32 to 36 completed weeks; and post-term is defined as ≥42 weeks.
The analyses were adjusted for the birth year of a child maternal age at child birth, parity (0 or ≥1), maternal cohabitation status at child birth (yes/no), maternal country of origin (Finland or other), maternal occupation (upper white-collar worker, lower white-collar worker, blue-collar worker, other status), smoking during pregnancy (yes/no), maternal obesity (*ICD-10* codes; E65-66, yes/no), maternal in-patient (from 1987) and out-patient (from 1998) psychiatric history (yes/no), maternal use of psychotropic medication during pregnancy (ATC codes; N05/N06, yes/no) and maternal systemic inflammatory disease (*ICD-10* codes M30-M36; yes/no).

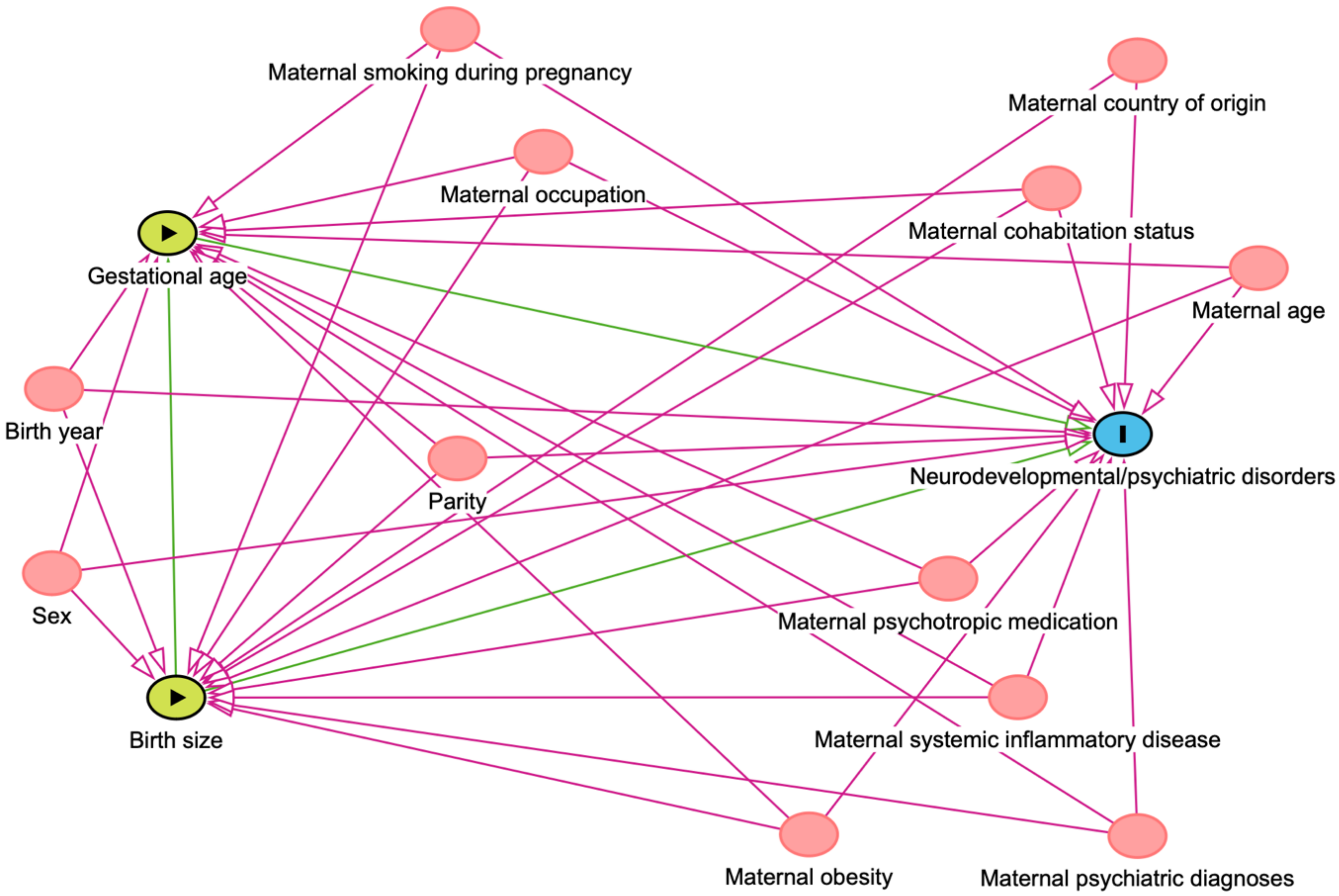

Fig S1. Directed acyclic graph representing the causal assumptions used for covariate selection based on existing literature, where nodes represent ancestors of exposures and/or outcomes, and arrows represent biasing or causal paths. Exposures are gestational age and birth size and outcome measures are neurodevelopmental and psychiatric disorders.

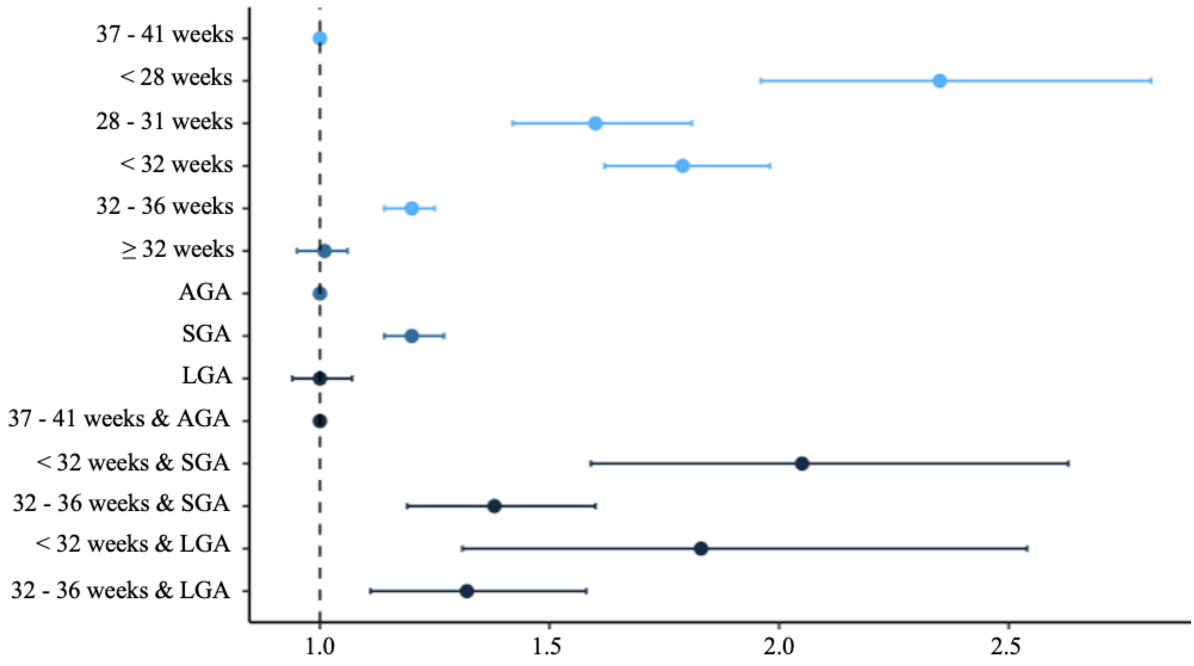

Fig S2. Adjusted hazard ratios (HRs) for feeding or eating disorders of childhood (F98.2-3), in relation to gestational age and size for gestational age (All live singleton spontaneous delivery births between 1996 and 2014 in Finland followed until 2018).

Abbreviations: AGA, appropriate for gestational age; SGA, small for gestational age; LGA, large for gestational age; SDD, specific developmental disorders; ASD, autism spectrum disorders; and ADHD, attention-deficit/hyperactivity disorders.

Extremely preterm is defined as < 28 weeks; very preterm is defined as 28 to 31 completed weeks; moderate-late preterm is defined as 32 to 36 completed weeks; term is defined as 37 to 41 completed weeks; and post-term is defined as ≥42 weeks.
